# Integrative Genomic Analysis of Cholangiocarcinoma Identifies Distinct *IDH*-Mutant Molecular Profiles

**DOI:** 10.1016/j.celrep.2017.02.033

**Published:** 2017-03-14

**Authors:** Farshad Farshidfar, Siyuan Zheng, Marie-Claude Gingras, Yulia Newton, Juliann Shih, A. Gordon Robertson, Toshinori Hinoue, Katherine A. Hoadley, Ewan A. Gibb, Jason Roszik, Kyle R. Covington, Chia-Chin Wu, Eve Shinbrot, Nicolas Stransky, Apurva Hegde, Ju Dong Yang, Ed Reznik, Sara Sadeghi, Chandra Sekhar Pedamallu, Akinyemi I. Ojesina, Julian M. Hess, J. Todd Auman, Suhn K. Rhie, Reanne Bowlby, Mitesh J. Borad, The Cancer Genome Atlas Network, Andrew X. Zhu, Josh M. Stuart, Chris Sander, Rehan Akbani, Andrew D. Cherniack, Vikram Deshpande, Taofic Mounajjed, Wai Chin Foo, Michael S. Torbenson, David E. Kleiner, Peter W. Laird, David A. Wheeler, Autumn J. McRee, Oliver F. Bathe, Jesper B. Andersen, Nabeel Bardeesy, Lewis R. Roberts, Lawrence N. Kwong

**Affiliations:** 1 Departments of Surgery and Oncology, Arnie Charbonneau Cancer Institute, University of Calgary, Calgary, AB T2N 4N1, Canada; 2 Departments of Genomic Medicine, Melanoma Medical Oncology, Bioinformatics and Computational Biology, Pathology, and Translational Molecular Pathology, The University of Texas MD Anderson Cancer Center, Houston, TX 77030, USA; 3 Human Genome Sequencing Center, Baylor College of Medicine, Houston, TX 77030, USA; 4 University of California Santa Cruz, Santa Cruz, CA 95064, USA; 5 The Eli and Edythe L. Broad Institute of Massachusetts Institute of Technology and Harvard University, Cambridge, MA 02142, USA; 6 Department of Medical Oncology, Dana-Farber Cancer Institute, Boston, MA 02215, USA; 7 Canada's Michael Smith Genome Sciences Centre, BC Cancer Agency, Vancouver, BC V5Z 4S6, Canada; 8 Center for Epigenetics, Van Andel Research Institute, Grand Rapids, MI 49503, USA; 9 Departments of Genetics and Pathology and Laboratory Medicine, University of North Carolina at Chapel Hill, Chapel Hill, NC 27599, USA; 10 Lineberger Comprehensive Cancer Center, University of North Carolina at Chapel Hill, Chapel Hill, NC 27599, USA; 11 Blueprint Medicines, 38 Sidney Street, Cambridge, MA 02139, USA; 12 Divisions of Gastroenterology and Hepatology and Laboratory Medicine and Pathology, Mayo Clinic College of Medicine, Rochester, MN 55905, USA; 13 Memorial Sloan Kettering Cancer Center, New York, NY 10005, USA; 14 University of Alabama at Birmingham, Birmingham, AL 35294, USA; 15 HudsonAlpha Institute for Biotechnology, Huntsville, AL 35806, USA; 16 USC/Norris Comprehensive Cancer Center, University of Southern California, Los Angeles, CA 90033, USA; 17 Division of Hematology and Oncology, Mayo Clinic, Scottsdale, AZ 85054, USA; 18 Departments of Hematology and Oncology, Massachusetts General Hospital, Harvard Medical School, Boston, MA 02114, USA; 19 Departments of Pathology and Oncology, Massachusetts General Hospital, Harvard Medical School, Boston, MA 02114, USA; 20 National Cancer Institute, Bethesda, MD 20892, USA; 21 Biotech Research and Innovation Centre, Department of Health and Medical Sciences, University of Copenhagen, Copenhagen 2200, Denmark

## Abstract

Cholangiocarcinoma (CCA) is an aggressive malignancy of the bile ducts, with poor prognosis and limited treatment options. Here, we describe the integrated analysis of somatic mutations, RNA expression, copy number, and DNA methylation by The Cancer Genome Atlas of a set of predominantly intrahepatic CCA cases and propose a molecular classification scheme. We identified an IDH mutant-enriched subtype with distinct molecular features including low expression of chromatin modifiers, elevated expression of mitochondrial genes, and increased mitochondrial DNA copy number. Leveraging the multi-platform data, we observed that ARID1A exhibited DNA hypermethylation and decreased expression in the IDH mutant subtype. More broadly, we found that IDH mutations are associated with an expanded histological spectrum of liver tumors with molecular features that stratify with CCA. Our studies reveal insights into the molecular pathogenesis and heterogeneity of cholangiocarcinoma and provide classification information of potential therapeutic significance.

## Graphical abstract

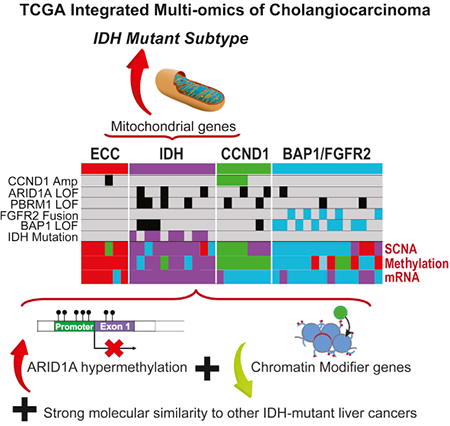

## Introduction

Cholangiocarcinomas (CCAs) are a group of malignancies of the biliary epithelium (cholangiocytes), comprising invasive carcinomas that arise in the intrahepatic, perihilar, and extrahepatic biliary tree (Razumilava and Gores, 2014). Most patients with CCA present with advanced disease and have a median survival of less than 1 year despite treatment with current standard chemotherapy (Valle et al., 2010). Even patients who undergo apparently curative resection have poor outcomes due to a high rate of tumor recurrence (Razumilava and Gores, 2014). Although intrahepatic, perihilar, and extrahepatic CCAs share morphologic features and have traditionally been aggregated in clinical trials, it is now apparent that there are important differences in tumor biology and genetics among tumors from different anatomic sites (Chan-On et al., 2013; Churi et al., 2014). Further elucidation of molecular alterations in these heterogeneous tumors and discovery of meaningful subtypes within each anatomic group are important steps toward developing more rational, specific, and effective treatments (Kelley and Bardeesy, 2015).

Cholangiocarcinoma is the second most common type of primary liver tumor, and its incidence has been rising in the United States, from 0.44 per 100,000 in 1973 to 1.18 in 2012 (Saha et al., 2016). The actual incidence of the disease is likely much higher, as recent gene expression studies suggest that “carcinomas-of-unknown primary” identified in the liver most commonly originate from biliary epithelium (Varadhachary and Raber, 2014). Worldwide, the highest incidence of CCA is found in northeastern Thailand, where parasitic infection by liver flukes (*Clonorchis sinensis* and *Opistorchis viverrini*) leads to infestation of the biliary tree (Razumilava and Gores, 2014). In regions without liver fluke infestation, CCA disease etiologies include: primary sclerosing cholangitis, hepatitis B or C virus (HBV/HCV) infection, biliary stone disease, congenital biliary malformations, cirrhosis, and exposure to aromatic toxins (Razumilava and Gores, 2014). Lifestyle-related factors such as smoking, alcohol consumption, and diabetes also contribute to the risk of intrahepatic CCA (iCCA) (Palmer and Patel, 2012). Given the diversity of risk factors influencing the mutational spectrum and the distinct cellular origins of the CCA subtypes, there is still an incomplete appreciation of the mechanisms of biliary carcinogenesis.

Prior studies indicate that iCCAs are unusual among epithelial cancers in having a relatively high rate of missense mutations in the isocitrate dehydrogenase 1 and 2 (*IDH1*/*IDH2*) genes (Borger et al., 2012; Chan-On et al., 2013; Jiao et al., 2013; Kipp et al., 2012; Ross et al., 2014; Wang et al., 2013), which encode metabolic enzymes that interconvert isocitrate and alpha-ketoglutarate in central carbon metabolism. These mutations, which are also common in acute myeloid leukemia, low-grade glioma and glioblastoma, and chondrosarcoma, occur at defined hotspots and result in neomorphic enzyme activity, leading to production of high levels of the metabolite (R)-2-hydroxyglutarate (2HG) (Losman and Kaelin, 2013). 2HG does not participate in normal metabolic processes but instead interferes with the function of enzymes that utilize alpha-ketoglutarate as a co-enzyme, including histone and DNA demethylases, and inhibits the mitochondrial electron transport chain (Fu et al., 2015; Parker and Metallo, 2015). Additional recurrent mutations and fusions have been reported in CCAs involving the fibroblast growth factor receptor 2 (*FGFR2*) gene, as well as in KRAS, BRAF, TP53, and in genes encoding chromatin-modifying enzymes (Borad et al., 2014; Churi et al., 2014; Jiao et al., 2013; Nakamura et al., 2015; Ross et al., 2014; Sia et al., 2015). Given this complexity, it is imperative to generate a more integrative model of the molecular alterations in iCCA to better define the oncogenic circuitry and to derive a classification system that groups tumors into biologically meaningful subtypes that can be used to guide therapy.

In this study, we describe molecular features of 38 liver fluke-negative CCAs—predominantly intrahepatic and hepatitis negative—that stratify the disease into distinct groups. Most notably, we identify a class of CCAs with distinct transcriptomic, copy number, and methylation profiles that are highly enriched for *IDH* mutant samples. We also identify pathways and methylation patterns that we validate in external datasets and which could help lead to the development of more effective treatments. Finally, cross-platform comparisons of CCA with pancreatic cancer and hepatocellular carcinoma (HCC) further emphasize the presence of distinct tumor subsets.

## Results

### Samples

We analyzed 38 CCA samples that were predominantly from North America (89%), intrahepatic (84%), fluke-negative (100%), and HBV/HCV-negative (97%, as assessed by combined DNA and RNA sequencing, which also revealed no HBV integration sites), and had no prior exposure to chemotherapy or radiation (Tables 1 and S1). This relative overall sample uniformity minimizes known and potential sources of molecular heterogeneity in our sample set.

The TCGA analysis pipeline used in this study consists of the following platforms: wholeexome sequencing; Affymetrix SNP 6.0 copy number array; RNA sequencing (RNA-seq), including microRNA (miRNA) and long noncoding RNA (IncRNA); DNA methylation; and reverse-phase protein array (RPPA) utilizing 192 antibodies. These datasets are annotated with relevant clinical information and careful histopathologic review by several experienced hepatobiliary pathologists (Table S1).

### Gene-Level Mutations, Fusions, and Copy Number Alterations

**Figure 1:**
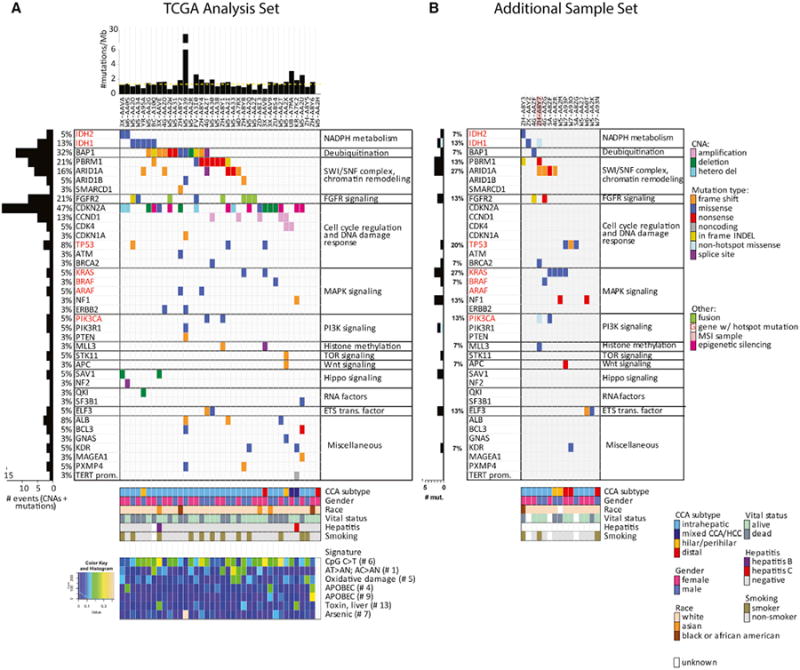
Somatic Alterations in Cholangiocarcinoma (A and B) Significantly mutated genes identified using the MutSigCV algorithm, and additional genes with chromosomal alterations, hotspot mutations (red font), and possibly functional mutations, grouped by pathway. (A) TCGA analysis sample set (n = 38). (B) Additional sample set (n = 15). Left, mutation amount and percentage, plus epigenetic silencing for CDKN2A. Top, overall number of mutations per megabase. Bottom, mutation spectra signatures. Dashed yellow line in upper panel indicates median mutations/megabase.

We first annotated alterations to specific genes. Whole-exome sequencing identified 2,831 somatic mutations, of which 1,869 (66%) were non-silent coding mutations. Targeted-capture, deep sequencing validation of 43 selected genes confirmed 77 mutations and newly identified nine (see Supplemental Experimental Procedures and Table S2). The median mutation rate was 1.38 per megabase, with a median mutation count of 49 (Figure 1A; Table S1). Compared to other cancers assessed by TCGA (Lawrence et al., 2013), this CCA mutation rate is intermediate, and comparable to that of pancreatic ductal adenocarcinoma (PDAC).

Consistent with previous studies, we identified inactivating mutations in the tumor suppressor genes *ARID1A*, *ARID1B*, *BAP1*, *PBRM1*, *TP53*, *STK11*, and *PTEN*, and hotspot gain-of-function mutations in the oncogenes *IDH1*, *IDH2*, *KRAS*, *BRAF*, and *PIK3CA* (Figures 1A and S1A; Tables S1 and S3). Also consistent with prior reports, the *IDH* mutant alleles described in our cohort (*IDH1*^R132C^ and *IDH2*^R172K/S^) are distinct from those found in glioma and acute myeloid leukemia (enriched for *IDH1*^R132H^ and *IDH2*^R140Q^) (Cancer Genome Atlas Research Network, 2013; Brat et al., 2015). In two tumors, we identified a recurrent P216L mutation in the regulatory domain of *ARAF* adjacent to the functionally validated N217I mutation (Sia et al., 2015), which suggests an activated state. We also identified two frameshift deletions and one missense mutation S217F (Zou et al., 2014) in the albumin gene (*ALB*), one of the most significantly mutated genes in HCC (Schulze et al., 2015). Finally, we detected a single telomerase reverse transcriptase (*TERT)* promoter mutation, in a mixed HCC/iCCA sample. To further validate the observed mutations, we performed whole-exome sequencing and targeted deep validation on an independent set of 15 samples (Figure 1B). Although analyzed separately, these additional samples corroborated the mutations above and highlighted additional recurrent mutations in the *BRCA2*, *MLL3*, *APC*, *NF1*, and *ELF3* tumor-suppressor genes.

An analysis of gene fusions from RNA-seq data identified five samples (13%) that expressed *FGFR2* fusion transcripts; this prevalence is consistent with other studies (Arai et al., 2014; Churi et al., 2014; Goyal et al., 2016; Nakamura et al., 2015; Ross et al., 2014). Our cases included two with the known fusion partner *BICC1* and three with the partners *KIAA1598*, *FRK*, and *C10ORF118* (Table S3). Other than the *FGFR2*-*FRK* fusion, which resulted in loss of the *FGFR2* kinase domain and retention of the *FRK* kinase domain, the rest of the fusions retained the kinase domain, consisting of *FGFR2* exons 1–17 spliced in frame with the partner gene. We note that *BICC1*, *KIAA1598*, and *C10ORF118* are located on chromosome 10 along with FGFR2. We also observed two missense mutations and one in-frame insertion in *FGFR2*.

Somatic copy number alterations (SCNAs) determined by analysis of high-density SNP data identified recurrent focal losses of *CDKN2A* and amplifications of *CCND1*. We further identified low-prevalence cases of focal SCNAs that have been reported in other cancers, including amplification of *CDK4/MDM2* and homozygous focal deletion of *QKI* and *SAV1* (Table S1; Figure S1B). The functional perturbation of these genes is supported by correlative gene expression data (Figure S1C). Epigenetic silencing of *CDKN2A* was identified in eight cases (21%) and was mutually exclusive with homozygous deletions and mutations (Figures 1, S1D, and S1E). Collectively, *CDKN2A* was mutated, deleted, or silenced in 47% of cancers, a higher rate than previously appreciated with single platform analyses.

Next, cross-comparing sequencing and copy number data, we found that all mutations in *BAP1* and *PBRM1* (both located on 3p21) were detected in tumors with 3p loss of heterozygosity, suggesting biallelic inactivation of these genes in near-diploid tumors. Cancer cell fractions were higher for the broad or arm-level loss of chromosome 3 than for *BAP1* mutations, followed by *PBRM1* mutations, suggesting that these events occur chronologically (3p loss, *BAP1*, *PBRM1*) in CCA development (Figures S1F and S1G). We also note that the NF2 splice site mutation in sample AA0S experienced loss of heterozygosity, suggesting bi-allelic loss of this Hippo pathway tumor suppressor gene.

Finally, using a previously defined mutational signature assessment (Covington et al., 2016) and non-negative matrix factorization, we identified seven enriched mutation signatures out of 21 total signatures. As observed across 31 tumor types analyzed to date (Covington et al., 2016), the most common signature was C > T/G > A substitutions at CpG islands (signature #6), followed by signature #1, characterized by AC > AN, AT > AN (Figure 1).

### Filtering Normal Liver Genes Uncovers an *IDH*-Mutant-Enriched mRNA Subgroup

We next analyzed mRNA expression by unsupervised hierarchical clustering, selecting only the most variable 2% (i.e., 400) of genes. The two resultant clusters showed a large differential expression of the genes (Figure S2A) with strong enrichment for a liver-associated signature. However, the clusters did not correlate with any other molecular or clinical parameter. Given that most liver signature genes are expressed in normal liver at levels 1,000- to20,000-fold higher than in tumors, we considered that the liver signature expression in part reflected contamination by even a small amount of normal liver. Consistent with this hypothesis, histological analysis revealed a trend toward higher normal liver contamination in the liver-high cluster (Figure S2C), which was not picked up by DNA-based tumor purity estimates. This indicates that high-expressing stromal genes likely confound mRNA expression clustering, particularly when using only the top-most variable genes.

**Figure 2:**
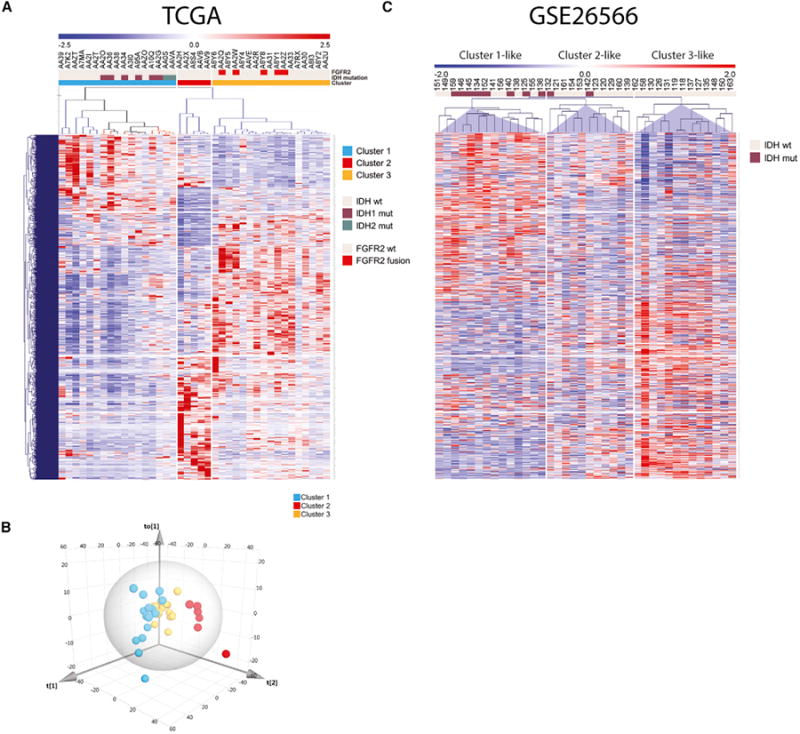
mRNA Analysis of Cholangiocarcinoma Identified an *IDH*-Enriched Cluster (A and B) Principal component analysis of RNA-seq expression data of 15,272 genes after exclusion of 541 normal liver genes. The heatmap in (A) shows the most strongly discriminant 973 genes (shared between the TCGA and the GSE26566 dataset) that define the three clusters. (B) Three-dimensional PCA plot of TCGA CCA samples. (C) Hierarchical clustering analysis of 40 samples from the CCA microarray dataset GSE26566, using the same 973 genes as in (A). Genes in heatmaps (A) and (C) are shown in the same order.

To reduce the statistical effects of the liver signature, 386 liver-specific genes derived from the GTEx (Genotype-Tissue Expression) normal tissue expression database (Lonsdale et al., 2013) (Table S4) were filtered out. The remaining 15,427 genes underwent principal component analysis (PCA) (Figures 2A and 2B), identifying three clusters. A subset of 1,150 genes was identified by orthogonal partial least-squares discriminant analysis (OPLSDA) as most strongly contributing to cluster separation (Figure 2A). Intriguingly, cluster 1 included all seven cases with an *IDH1* or *IDH2* hotspot mutation, while cluster 2 was enriched in extrahepatic or perihilar CCA, and cluster 3 contained all five *FGFR2* fusions. This was validated by comparison with the previously generated microarray gene expression dataset GSE26566 (Andersen et al., 2012; Wang et al., 2013). Hierarchical clustering was performed for the 976 most strongly discriminant genes shared between datasets (Figure 2C). In this validation cohort of 40 samples, three main clusters were identified that resembled the TCGA dataset clusters. Notably, most *IDH1*/2 mutations (eight of ten) were located in the cluster that most strongly resembled the *IDH*-mutant-enriched TCGA cluster 1. Thus, removal of the liver signature unmasked transcriptional clusters that segregated samples in a biologically relevant manner.

### The *IDH* Mutant Subgroup Is Enriched for Mitochondrial and Chromatin-Modifier Signatures

**Figure 3:**
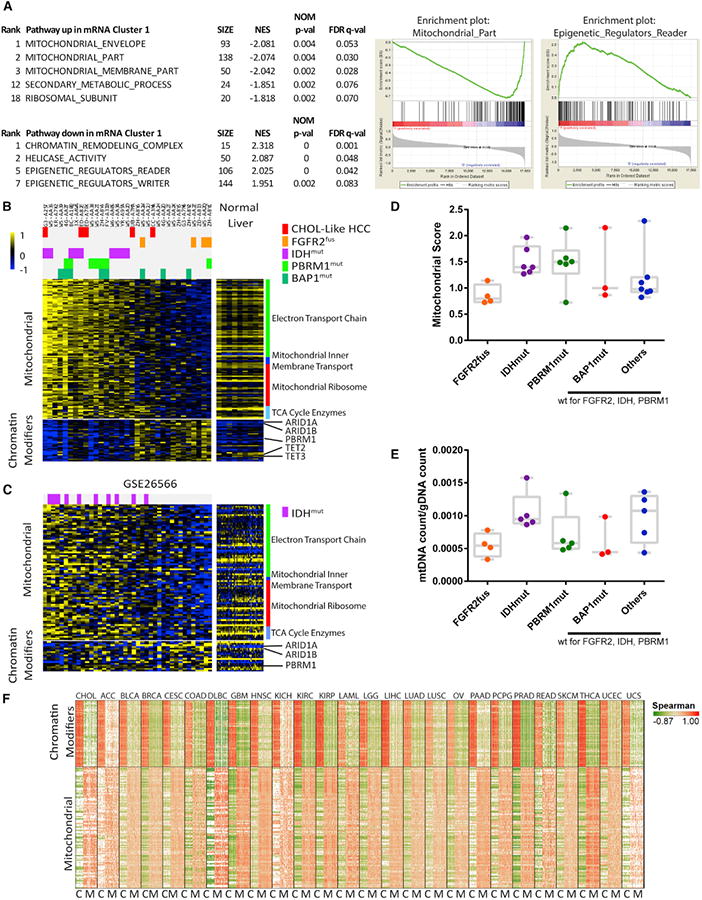
*IDH* Mutant Cancers Are Enriched for a High Mitochondrial Signature and Mitochondrial DNA Count and a Low Chromatin Modifier Signature (A) GSEA analysis identified mitochondrial and chromatin modifier genes as significantly differentially expressed in the *IDH*-mutant-enriched mRNA cluster 1. Selected pathways are shown, omitting genesets that show a high degree of gene membership overlap with the displayed pathways. Full results are available in Table S6. NES, normalized enrichment score; size, geneset size. (B and C) Heatmaps of the most significant mitochondrial and chromatin-modifier genes for TCGA (B) and GSE26566 (C). TCGA samples are filtered for high purity (>0.65); unfiltered results are shown in Figure S9. (D and E) Quantification of mitochondrial signature (D) and mitochondrial DNA (E) count for different mutational subgroups, showing high-purity samples only. No subgroup by itself is significantly different from all other samples, indicating only enrichments and not exclusivity for high/low mitochondrial markers. Box and whisker plots show maximum and minimum bars. (F) Pan-cancer correlation analysis of mitochondrial and chromatin modifier genes. For each cancer, the genes on the x and y axes are the same and in the same order. Red signifies high positive Spearman correlation values; green denotes high negative values for each gene-gene comparison. C, chromatin modifiers; M, mitochondrial genes. Genes are listed in Table S8.

We next performed gene set enrichment analysis (GSEA) on the mRNA clusters and discovered an enriched expression of genes involved in mitochondrial structure and function in the *IDH*-mutant-enriched cluster 1. This included notable upregulation of genes encoding citric acid cycle enzymes, mitochondrial ribosomal proteins, electron transport chain components, and mitochondrial structural constituents, consistent with altered control of oxidative phosphorylation and mitochondrial biogenesis (Figure 3A; Tables S5 and S6) (hereafter collectively referred to as “mitochondrial gene expression”). High mitochondrial gene expression was significantly associated with *IDH* and *PBRM1* mutant samples and low expression with *FGFR2*-fusion samples (Figure 3B and depicted as a condensed “mitochondrial score” in Figure 3D); these correlations were improved by removing low-purity (<0.65) samples, a possible confounding factor (Figures S3A and S3B). In keeping with potential functional relevance of the differential expression of mitochondrial genes, we identified a relatively higher mitochondrial copy number (Reznik et al., 2016) in *IDH* mutant samples and a lower number in *FGFR2*-fusion samples (Figure 3E).

Examination of the GSE26566 dataset (Andersen et al., 2012) provided an external validation of these findings, again identifying an enrichment of *IDH* mutants among the tumors with high expression of the mitochondrial gene signature (Figure 3C). This association between this signature and *IDH* mutations appears to be particular to CCA, since it was not observed upon analysis of TCGA datasets for glioblastoma, low-grade glioma, melanoma, or acute myeloid leukemia TCGA datasets (Figure S3C).

GSEA also identified chromatin modifier gene sets as significantly downregulated in the *IDH* mutant mRNA cluster (Figure 3A; Tables S5, S6, and S7. Notably, these included genes recurrently mutated in CCA—*ARID1A*, *ARID1B*, and *PBRM*—as well as genes whose protein products are known to be inhibited by *IDH*-mutant-generated 2HG, including *TET2*, *TET3*, *KDM2A*, and *KDM5B* (Xu et al., 2011). Expression of the chromatin modifier geneset anticorrelated with the mitochondrial geneset even when considered as a gradient (Figure S3D; Table S7). Querying of this association in multiple TCGA gene expression datasets demonstrated strikingly consistent anticorrelation of these two pathways across 23 of 25 cancer types (Figure 3F) as well as across and within normal tissues (Figure S3E) from the GTEx database for most genes. These results suggest that mitochondrial activity and chromatin modification are linked basic biological events that are also regulated by *IDH* hotspot mutations in CCA.

### Cluster-of-Clusters Analysis Identifies Four Subgroups

**Figure 4:**
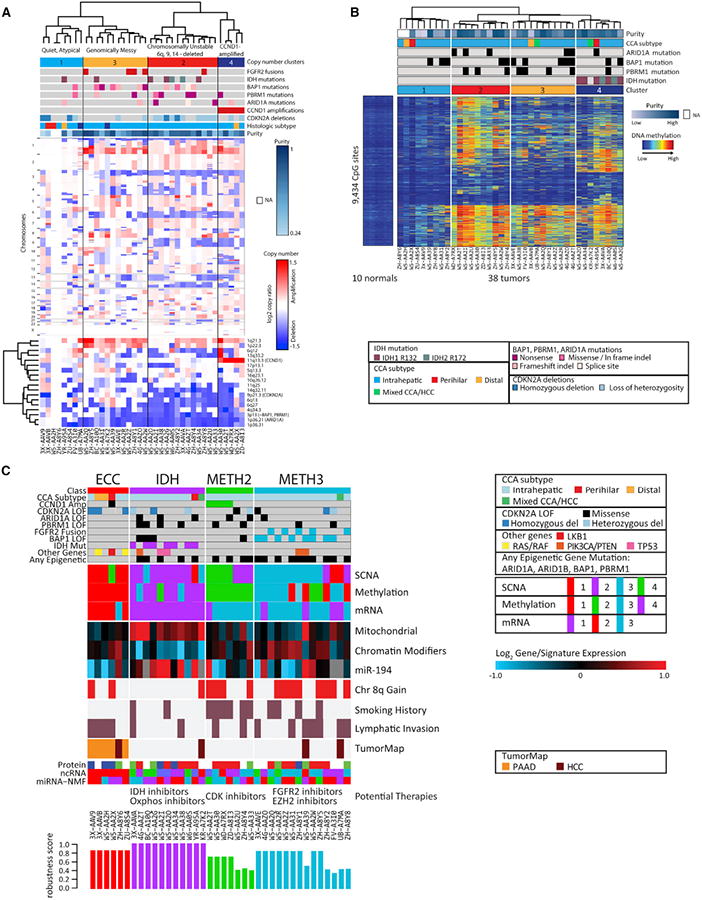
Cluster-of-Clusters Analysis of CCAs (A) Unsupervised hierarchical clustering of copy number data. The clustering is performed on SCNAs that are determined by GISTIC analysis to be significantly altered, as shown in the lower heatmap. (B) Unsupervised hierarchical clustering of DNA methylation data. (C) The cluster-of-clusters analysis (COCA) was performed on the three platforms with the strongest degree of correlation (mRNA, copy number, and methyl-ation). Bottom, robustness scores indicating strength of cluster membership assignment for each sample. Selected information of interest is shown here; full clustering, genetic, clinical, and pathological data are available in Table S1.

We next enlisted the additional platforms (copy number, methylation, miRNA, IncRNA, and protein) into a clustering of the cluster assignments (COCAs) (Hoadley et al., 2014), which provides a way to distinguish sample subtypes by identifying patterns across platforms. We started by performing hierarchical clustering of each platform separately. First, copy number based on SNP array hybridization data (Figure 4A) revealed four genomic clusters. We note that this clustering is performed on select SCNAs determined by genome identification of significant targets in cancer (GISTIC) analysis to be significantly differentially altered, as using all data points results in overfitting. This approach therefore highlights samples that share loci that are likely undergoing positive selection in the tumor. Cluster 4 consisted entirely of tumors with high-level amplification of *CCND1*. Cluster 2 was characterized by enrichment of chromosomal deletions, particularly 6q, 9, and 14. Cluster 3 tumors contained mostly SCNAs that were found across the larger set of tumors (e.g., 1p loss and 1q gain), but on average had fewer arm-level deletions than cluster 2. Last, cluster 1 consisted of molecularly atypical tumors, including two genomically silent cases that were completely devoid of copy number alterations or recurrent CCA driver mutations (the low-purity extrahepatic W5-AAH2 and the 0.61-purity intrahepatic ZH-A8Y6).

Next, unsupervised clustering of samples using CpG sites that show cancer-specific DNA methylation changes identified four subgroups in our CCA cohort (Figure 4B). Tumors in cluster 1 showed minimal alterations in DNA methylation compared with normal liver, which is at least partially explained by low tumor purity for most of the samples. The remaining tumors had prominent DNA hypermethylation and were classified into three subgroups. All seven *IDH* mutant tumors were present in cluster 4, along with one *IDH*-wild-type case that exhibited a gene expression profile similar to that of *IDH* mutants (see Figure S4A). Surprisingly, on average, tumors in cluster 2 showed an even greater degree of DNA hypermethylation than did *IDH* mutant tumors (Figures 4B and S4B). Last, we note that tumors in clusters 2 and 3 had frequent mutation of genes encoding chromatin regulators, including *PBRM1* and *ARID1A* (ten of 20).

We observed that the copy number and methylation clusters generally matched the mRNA clusters identified in Figure 3, suggesting the ability of the data to detect shared biological mechanisms. By contrast, hierarchical clustering of mature miRNAs, IncRNAs, and protein yielded clusters that were discordant with the other platforms. We believe this is mainly due to the far smaller number of informative features available for clustering (169, 101, and 192, respectively) given the sample size (Figure S4). As expected, COCA analysis using all six platforms gave a discordant pattern with no clinical correlates, even when the lower-sample number RPPA was removed. We therefore conducted the COCA analysis using only mRNA, copy number, and methylation to discern biologically coherent clusters. We optimized a four-cluster solution (see Experimental Procedures) that was not dominated by any one platform (Figure 4C).

We then correlated clinical data and molecular aberrations with the four COCA clusters (Figure 4C; Table S1). *IDH* hotspot mutations were present exclusively in COCA2 (p = 0.0004; “IDH COCA”), reflecting the mRNA and methylation specificity noted earlier, and identifying a correlation with copy number cluster 2 (“genomically unstable”). Patients with IDH COCA tumors were typically nonsmokers, and the tumors exhibited a lower frequency of lymphatic invasion and chromosome arm 8p gains (Figure 4C). Three of four distal or hilar tumors were in COCA1 (p = 0.003; “ECC COCA”), which exhibited the following characteristics: wild-type for *FGFR2*, *IDH1*/2, *ARID1A*, *BAP1*, and *PBRM1*; low methylation; and relative genomic silence for copy number alterations. COCA3 was enriched for samples with *CCND1* amplification and with the most highly hypermethylated profile (methylation cluster 2; “METH2 COCA”). COCA4 (“METH3 COCA” contained eight of 12 cases with *BAP1* mutations (p = 0.01) and all five *FGFR2* fusion cases (p = 0.004). Survival analysis among the COCA clusters did not yield significant p values, possibly due to the small sample size. All clustering solutions for individual platforms and for COCAs, as well as key genetic, clinical, and pathologic data are available in Table S1. We posit that these COCAs identify biologically distinct CCA subtypes with potential clinical implications; however, we also acknowledge the limitations due to the sample number, and that validation of these subtypes awaits new comparable datasets and functional confirmation in model systems. Nevertheless, these results clearly highlight the molecular distinctness of *IDH* mutants and the power of integrated multiplatform analyses.

### ARID1A Promoter Hypermethylation and Decreased Expression in *IDH* Mutants

**Figure 5:**
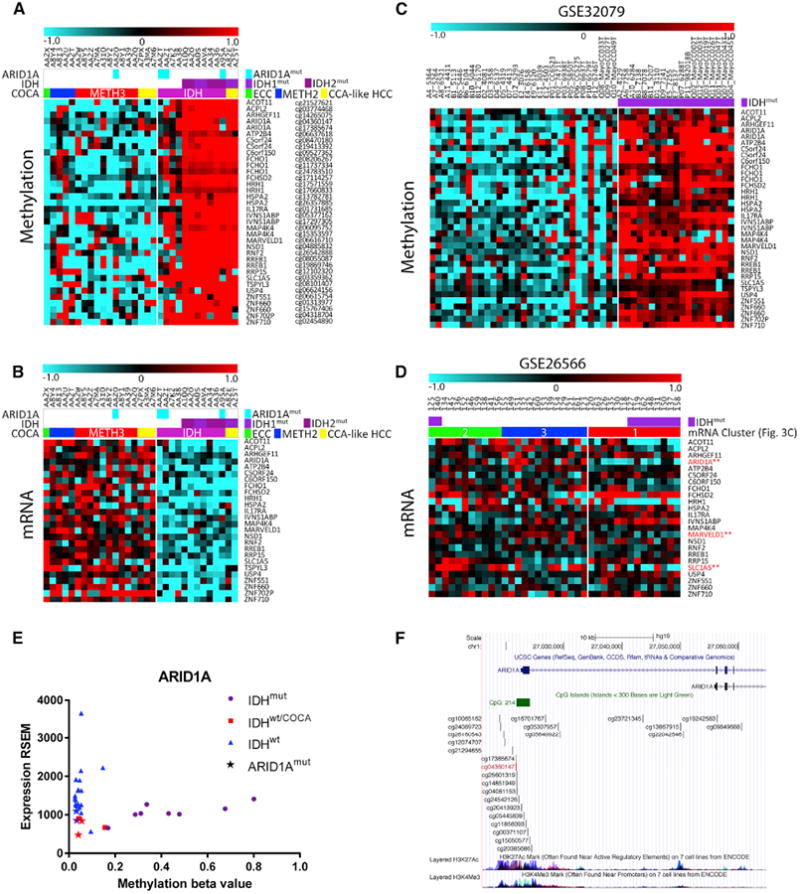
ARID1A Is Hypermethylated and Has Low Expression in the IDH COCA (A and B) TCGA methylation (A) and RNA-seq (B) data for 24 genes (36 probes) that show both IDH^mut^-specific hypermethylation and downregulation in the IDH COCA subtype. (C and D) Methylation (C) and microarray (D) data for the same 24 genes and 36 methylation probes as in (A and B), in the publically available datasets GEO: GSE32079 and GSE26566. (E) Scatterplot of TCGA methylation and RNA expression values for ARID1A. (F) Location of the two ARID1A hypermethylated probes within the ARID1A promoter. ENCODE histone marks for H3K27Ac and H3K4me3 are shown.

To extend our analysis of the *IDH* mutant subgroup, we considered that the COCA classifications may help reveal new cross-platform insights. To this end, we asked whether *IDH*-mutant-specific DNA hypermethylation may target genes that show decreased expression in the IDH COCA subtype. We restricted the analysis to high-purity samples to avoid assessing gene expression changes that are primarily associated with contaminating stroma rather than with methylation. After intersecting IDH mutant hypermethylated loci with genes with decreased expression in the IDH COCA subtype and filtering for gene-specific anticorrelation between the two platforms, we identified a list of 24 genes whose expression is putatively regulated by *IDH* mutant hypermethylation (Figures 5A and 5B). To validate this list, we cross-referenced it with the matched CCA methylation and expression datasets GSE32079 and GSE26566, respectively (Andersen et al., 2012; Wang et al., 2013). GSE32079 uses the same methylation array platform as the TCGA dataset, allowing for direct comparison of probes. While all 24 genes once again showed *IDH*-mutant-specific hypermethylation (Figure 5C), only three genes, ARID1A, MARVELD1, and SLC1A5 were significantly downregulated (Figure 5D) in the “IDH-like” mRNA cluster (cf. Figure 2C). Given the bona fide tumor suppressor role of ARID1A in the liver, we explored the relationship between DNA methylation and expression further (Figure 5E). Of four *IDH-*wt samples in the IDH COCA, two were ARID1A mutant with low ARID1A expression. Moreover, the only IDH mutant to not show ARID1A hypermethylation, A95A, was ARID1A mutant with low expression (Figures 5A and 5E), suggesting that ARID1A mutation and IDH-induced hypermethylation are mutually exclusive due to redundancy. Analysis of publically available histone modification ChIP-seq data showed that the two hypermethylated ARID1A probes were located in the ARID1A promoter within the active transcription marks H3K27Ac and H3K4me3 (Figure 5F). Collectively, these data suggest that *IDH* mutations result in hypermethylation and silencing of ARID1A, and that impingement of ARID1A is a convergent feature of IDH COCA tumors.

### IncRNAs Associated with the Chromatin Modifier Signature

Given the centrality of the chromatin modifier signature to the IDH COCA subtype, we explored the remaining platforms to identify non-mRNA members of the signature. As IncRNAs are relatively understudied, we sought to identify IncRNAs that correlated with the mRNA-based chromatin modifier signature. To further limit the resulting set of 66 IncRNAs, we asked which ones also tracked with the chromatin modifier signature across other TCGA datasets (cf. Figure 3F). Across eight assessed cancers, we found that 21 of the IncRNAs correlated with the chromatin modifier signature in at least six cancers (Figure S5A; Table S7). Importantly, only an estimated eight of the 66 IncRNAs were expected to correlate by chance, making 21 a significant enrichment (p = 0.01, Fisher's exact test). These findings suggest potential functions for these 21 IncRNAs, which have not previously been studied.

Additionally, we identified IncRNA clusters that correlate with immune and liver mRNA signatures (Figures S5B and S5C) in the CCA dataset. To validate these, we determined the overlap with high-stringency immune- and liver-specific IncRNAs defined from the GTEx database (Lonsdale et al., 2013): for immune-associated IncRNAs, 34/48 CCA-derived IncRNAs overlapped with 190 GTEx IncRNAs (p = 2 × 10^-13^), while for liver-associated IncRNAs, 25/25 CCA-derived IncRNAs overlapped with 127 GTEx IncRNAs (p = 4 ×10^-13^). These clusters lend support to the biological fidelity and analytical utility of the IncRNA platform and provide starting IncRNA candidates when analyzing future samples. For miRNAs, we note that miRNA-194-5p is significantly upregulated in the IDH COCA subtype and negatively correlated with the chromatin modifier signature (Figures 4 and S5D–S5H). Notably, miRNA-194 has been implicated as a suppressor of invasion in liver cancer in vitro (Meng et al., 2010). The results of these analyses collectively demonstrate the robustness of the pan-cancer and cross-platform capacity of the TCGA.

### Comparison with HCC and PDAC

We next compared liver, pancreatic, and biliary cancer across the standardized multiplatform TCGA datasets to determine their molecular relationships (Borad et al., 2014). First, to improve comparisons of mutational landscapes, we conducted a meta-analysis of six iCCA sequencing studies (Churi et al., 2014; Jiao et al., 2013; Nakamura et al., 2015; Ruzzenente et al., 2016; Zou et al., 2014) including this one, totaling 458 samples (Table S8). Whereas PDAC is dominated by *KRAS* (92%) and *TP53* (70%) mutations, *KRAS/NRAS* and *TP53* mutations comprise only 20% and 21% of iCCAs, respectively. HCC is characterized by *TERT* promoter (46%) and *CTNNB1* (26%) mutations, neither of which are present in iCCA; otherwise HCC shares only three genes with iCCA that are mutated at >5% (*TP53*, *BAP1*, and *ARID1A)*.

**Figure 6:**
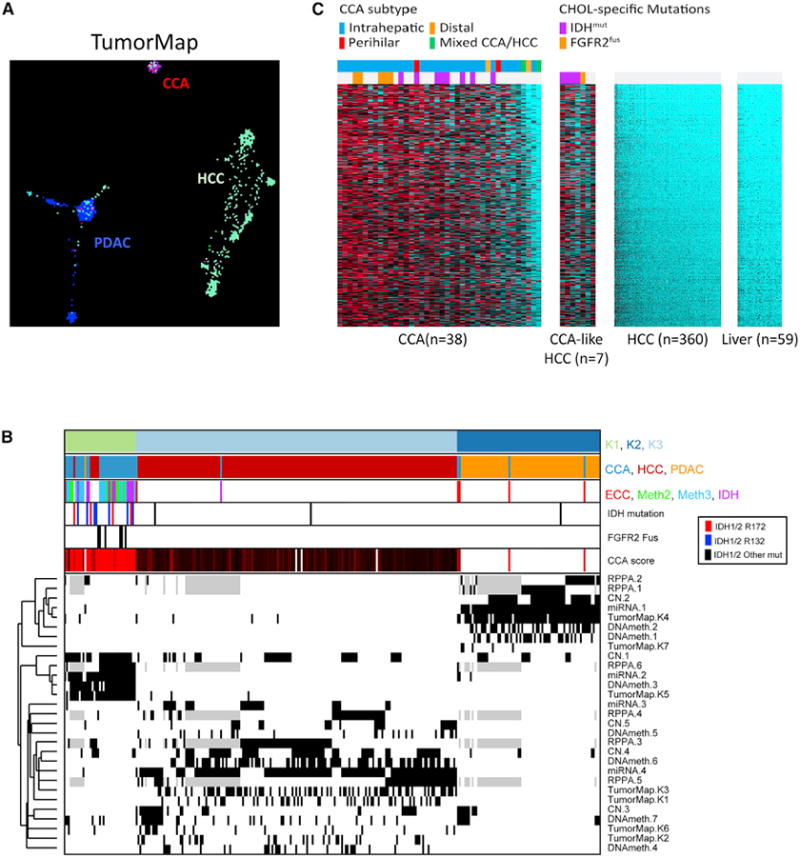
Cross-Cancer Analysis Comparing TCGA Cholangiocarcinoma, HCC, and Pancreatic Adenocarcinoma (A) Tumor map analysis incorporating mRNA, methylation, and copy number showing proximity of each sample. (B) COCA across miRNA, copy number, DNA methylation, RPPA, and Tumor Map for the three cancer types. Unsupervised clustering was performed within each data type across a cohort of 292 samples from CCA, HCC, and PDAC to derive cross-tumor subtypes (miRNA n = 4; copy number (CN) n = 5; DNA methylation (DNAmeth) n = 7; RPPA n = 6; tumor map n = 7; see Figure S6 for individual platform cluster solutions). Individual classification subtypes were then used as input for pan-tumor COCA analysis identifying three COCA classes (first bar; K1, light green; K2, dark blue; K3, light blue). Second annotation bar denotes histology type – CCA, HCCC, PDAC. Third bar indicates the CCA-specific subtype classification (ECC, METH2, Meth3, and IDH, cf. Figure 5). Fourth bar notes IDH1 mutation status (red, R172 mutations; blue, R132 mutations; black, other mutations). Fifth bar indicates samples with FGFR2 fusions. Sixth bar indicates CCA score, a median value of the 600 most-enriched genes in CCA (see C). The bottom heatmap indicates sample membership for each of the individual classification subtypes (black, subtype member; white, not a subtype member; gray, missing data). Each row is labeled by platform and subtype number. (C) Six hundred genes enriched in cholangiocarcinoma-like HCC.

We next applied the Tumor Map algorithm, which generates “islands” of cancers based on similarity within chosen platforms (Davis et al., 2014). Incorporating mRNA expression, copy number, and methylation data (Figure 6A), this analysis separated most HCC, PDAC, and CCA samples into their respective cancer-type islands; however, seven of 38 (18%) CCA samples were embedded in the PDAC and HCC islands, and seven of 179 (4%) HCC samples were embedded in the CCA island, suggesting that some samples have discordant histopathologic and molecular profiles.

To better understand these discordances, we illustrated cluster memberships for all 292 CCA/HCC/PDAC samples (Table S9) after clustering within each molecular platform (mRNA, miRNA, RPPA, copy number, and methylation; Figures S6A–S6F). First, we noted that most of our COCA1 CCA samples (distal/hilar CCA-enriched) clustered with PDAC, consistent with the related developmental origins of the extrahepatic and pancreatic ducts from the foregut endoderm. Second, we studied in greater depth the seven HCCs that mapped with CCA. These tumors shared several molecular features with CCA, including mRNA and miRNA expression patterns, DNA methylation, and to a lesser extent copy number (Figure 6B). Moreover, they lacked *TERT* promoter mutations, which are a hallmark of HCC but are absent in CCA. Strikingly, five of those seven samples harbored either hotspot *IDH1*/2 mutations (n = 4) or an *FGFR2* fusion (n = 1), and they were the only cases in the HCC dataset with these mutations. Re-examination of their histology revealed that although regions of these seven cases fall within the spectrum of HCC, each of the five tumors with *IDH1* or *FGFR2* lesions had some features that have also been described in iCCA, including focal to diffuse glandular differentiation, abundant fibrotic stroma (desmoplasia), and in some areas, an anastomosing architecture (Figures S6G–S6L) (Bledsoe et al., 2015; Liau et al., 2014; Nakanuma et al., 2012). Consistent with this, these samples expressed bile duct (e.g., SOX9) and hepatocellular (e.g., HNF4A, HNF1A) markers at levels within that of iCCA (Figures 6C, S6M, and S6N). Analysis of the 600 genes that are most enriched in these tumors compared with standard HCC corroborated this close transcriptional similarity to CCA (Figure 6C). These data are notable in view of accumulating evidence that CCA and HCC lie along a spectrum of primary liver carcinomas, with intermediate subsets exhibiting overlapping phenotypes. The prominent enrichment of *IDH* mutations in molecularly CCA-like HCCs is consistent with previous findings that *IDH* mutations block liver progenitor cells from undergoing hepatocyte differentiation and shift them toward a cholangiocellular fate (Saha et al., 2014).

## Discussion

Taking advantage of the molecular resolution provided by multiple genomic platforms, we identified distinct mRNA, DNA methylation, and copy number subgroups that together specify biologically relevant CCA classes. In particular, we highlight an *IDH*-mutant-enriched class whose samples share similar profiles across these three platforms. Notably, this class exhibits high expression of mitochondrial genes, including components of the citric acid cycle and electron transport chain, accompanied by relatively high mitochondrial DNA copy number, as well as low expression of chromatin modifier genes. This anticorrelation of mitochondrial and chromatin modifier signatures appears to be a basic biological link spanning nearly all TCGA cancers and normal GTEx tissues analyzed (Figures 2F and S9), warranting deeper mechanistic studies. The anticorrelation is consistent with the hypothesis that global changes in histone acetylation and DNA methylation rates affect mitochondrial metabolism via imbalances in available pools of acetyl and methyl moieties (Martinez-Pastor et al., 2013). Relevantly, *IDH* mutants hypermethylate and putatively silence the ARID1A promoter, which may contribute to the lowered chromatin modifier signature expression.

Moreover, we identify a group of liver tumors with an atypical histopathology and a highly CCA-like molecular profile that is enriched for *IDH* mutations, consistent with the emerging view that liver tumors comprise a continuous spectrum (Fan et al., 2012; Holczbauer et al., 2013; Marquardt et al., 2015; Sekiya and Suzuki, 2012). Given the molecular and partial histologic similarity to CCA, this potential subtype may be a distinct clinical entity and strongly warrants further study into its most beneficial classification. Furthermore, the complete lack of *IDH* mutations in otherwise standard HCC from the TCGA set (0/172) has implications about specific functions of mutant *IDH* in modulating liver cell identity and also underscores the benefit of combined molecular and histopathological diagnosis. Although previous studies also identified transcriptionally CCA-like HCC (Oishi et al., 2012; Seok et al., 2012; Woo et al., 2010), our results identify *IDH* and *FGFR2* perturbations as associated drivers linked to methylation, miRNA, and copy number similarities. Together, these findings highlight the uniqueness of *IDH*-mutant-driven cancers and the importance of defective chromatin regulation in the pathogenesis of CCA.

Improved molecular classification of cholangiocarcinoma is urgently needed, as heterogeneity presents a serious challenge to clinical management. Unlike cancers in which a few predominant oncogenic loci converge on a pathway, such as *KRAS* in PDAC or the mostly mutually exclusive *BRAF*, *NRAS*, and *NF1* in melanoma, CCA is marked by a heterogeneous set of often-overlapping, lower-penetrance driver genes across diverse signaling pathways. This intertumoral heterogeneity is further exacerbated by geographically distinct molecular profiles and is modified by the presence or absence of liver fluke infestation and/or viral hepatitis, and by the anatomic location of the cancer. As examples, (1) extrahepatic CCAs have more *SMAD4* mutations than iCCAs (Churi et al., 2014; Ong et al., 2012); (2) a Chinese study found a much lower incidence of *IDH* (5%), *PBRM1* (1%), and *BAP1* (1%) mutations (Zou et al., 2014) in iCCAs; and (3) liver fluke- and/or viral hepatitis-positive cancers have a higher incidence of *TP53* mutations and lower incidence of *IDH* mutations (Chan-On et al., 2013; Ong et al., 2012; Zou et al., 2014). In this study, we focused on intrahepatic and fluke- and hepatitis-negative CCA, which minimized heterogeneity and improved the ability to apply categorizing statistics. Although larger studies are needed to validate and identify further molecular subclasses, our results provide a proof of principle that subclasses of CCAs have distinct multi-level molecular characteristics that suggest potential therapeutic approaches.

In this regard, the enriched mitochondrial gene signature and coordinate increase in mitochondrial number in *IDH* mutant CCA is intriguing in light of prior work implicating mutant *IDH* in the impairment of multiple aspects of cell metabolism (Cuyàs et al., 2015; Grassian et al., 2014; Tateishi et al., 2015). These observations include 2HG-mediated disruption of components of the mitochondrial electron transport chain, and inhibition of reductive glutamine metabolism, a wild-type *IDH1* -dependent process that is important for fatty acid synthesis in cells with dysfunctional mitochondria (Fu et al., 2015; Grassian et al., 2014; Leonardi et al., 2012). The *IDH* mutant iCCA profile might thus reflect an adaptive response to mitochondrial dysfunction and/or an increased reliance on mitochondrial activity for tumor growth. The decrease in expression of chromatin regulators is also notable given the potential widespread effects of 2HG on epigenetic states via inhibition of TET family cytosine oxygenases and Jumonji domain family histone demethylases (Xu et al., 2011). However, the significance of this signature is difficult to interpret, since the genes contributing to the signature spanned all classes of chromatin regulators as well as 21 newly associated IncRNAs. Nevertheless, the discovery of distinct molecular features of *IDH* mutant iCCA is noteworthy in light of early clinical data using *IDH1* -mutant-specific inhibitor (Burris et al., 2015). While promising, these data suggest that single-agent treatment with these drugs may not be sufficient to produce durable responses or remissions. Thus, targeting aspects of metabolism (e.g., using inhibitors of oxidative phosphorylation) or of chromatin regulation are tentatively suggested by our genomic findings as avenues for future research. Notably, prior work has suggested that *BAP1*, *PBRM1*, and *ARID1A* deficiency all result in sensitivity to EZH2 inhibition across cancer types (Kim et al., 2015; LaFave et al., 2015); the association of ARID1A methylation with IDH mutation opens the question of whether EZH2 inhibition might also be effective in this subtype. Collectively, our findings reveal distinct molecular characteristics of *IDH* mutant cholangiocarcinoma, offering insights and valuable multi-omics data as a springboard for future basic and translational research into this deadly disease.

## Experimental Procedures

Cholangiocarcinoma (CCA) tumors were collected and shipped to a central Biospecimen Core Resource (BCR) between August 15, 2013 and January 20, 2014. Qualifying tumor samples were obtained from patients who had received no prior chemotherapy or radiotherapy treatment for their disease. Specimens were shipped overnight from 12 Tissue Source Sites (TSSs) using a cryoport that maintained an average temperature of less than **—** 180°C. TSSs contributing biospecimens included Barretos Cancer Hospital (Barretos, Brazil); Emory University (Atlanta, GA, USA); Garvan Institute of Medical Research (Darlinghurst, NSW, Australia); ILSbio, LLC (Chestertown, MD, USA); Mayo Clinic (Rochester, MN, USA); Sapienza University of Rome (Rome, Italy); Spectrum Health (Grand Rapids, MI, USA); University of Calgary Alberta Health Services (Calgary, AB, Canada); University of California - San Francisco (San Francisco, CA, USA); University of New Mexico (Albuquerque, NM, USA); University of North Carolina (Chapel Hill, NC, USA); Wake Forest University (Winston-Salem, NC, USA).

In addition to tumor samples, each frozen primary tumor specimen had a companion normal tissue specimen (blood or blood components, including DNA extracted at the TSS). Adjacent nontumor tissue was also submitted for a subset of cases (n = 20).

Cases were staged according to the American Joint Committee on Cancer (AJCC) staging system. Pathology quality control was performed on each tumor specimen and on adjacent normal tissue (where available) from a frozen section slide prepared either by the BCR or by the TSS. H&E-stained sections from each sample were made available on to a team of independent pathologists for review to: confirm consistency with CCA histology, confirm that the adjacent tissue specimen contained no tumor cells, and annotate various standard pathological parameters. Only tumor samples with ≥60% tumor nuclei, and %20% necrosis were submitted for nucleic acid extraction.

The data and analysis results can be explored through the TCGA Data Portal (https://gdc-portal.nci.nih.gov/projects/TCGA-CHOL), the Broad Institute GDAC FireBrowse portal (http://firebrowse.org/?cohort=CHOL), and Memorial Sloan Kettering Cancer Center cBioPortal (http://www.cbioportal.org/study.do?cancer_study_id=chol_tcga#summary). Detailed experimental procedures are included in the Supplemental Experimental Procedures.

## Supplementary Material

Supplemental Experimental Procedures

Tables S1-S9.

## Figures and Tables

**Table 1:** Patient Characteristics

Characteristics	Number of Patients
Total number of patients	38
Median age at diagnosis (years)	66 (range 29–82)
Gender	
Female	21
Male	17
ECOG PS	
0	19
1	9
2	0
3	1
Unknown	9
Histologic Grade	
G1	5
G2	21
G3	9
G4	2
Unknown	1
Resection status (R0/R1/Rx)	28/7/3
Tumor Stage	
T1	19
T2 (T2a/T2b)	15 (2/5)
T3	4
Histologic Diagnosis	
Intrahepatic	32
Extrahepatic/hilar	4
Mixed ICC/HCC	2
Lymph node status (N0/N1/Nx)	26/5/7
Metastatic disease (M0/M1/Mx)	30/4/4
Lymphovascular invasion (yes/no/unknown)	15/22/1
Perineural invasion (yes/no/unknown)	4/33/1
Elevated CA 19-9 (yes/no/unknown)	18/13/7
Race (white/Asian/black)	33/3/2
Country submitting tumor (USA/Canada/Italy/Brazil/Vietnam	30/4/2/1/1
Elevated CA 19-9 (yes/no/unknown)	18/13/7
Presence of fluke infection (Y/N)	0/38

ECOG, Eastern Cooperative Oncology Group.

## Highlights

- IDH mutant CCAs have distinct mRNA, copy number, and DNA methylation features
- IDH mutant CCAs display high mitochondrial and low chromatin modifier gene expression
- IDH mutant CCAs methylate the ARID1A promoter and show low ARID1A expression
- Other *IDH* mutant liver cancers show multiplatform similarities to CCA

## In Brief

Farshidfar et al. present The Cancer Genome Atlas (TCGA) marker analysis of cholangiocarcinoma. Through multiplatform analyses, they identify a distinct subtype enriched for IDH mutants. This subtype shows increased mitochondrial and decreased chromatin modifier gene expression, including potential epigenetic silencing of ARID1A. Other IDH-mutant liver cancers molecularly resemble cholangiocarcinoma.
