# Supplementary material for: Integrative Genomic Analysis of Cholangiocarcinoma Identifies Distinct IDH-Mutant Molecular Profiles
Source: Cell Rep. Author manuscript; Available in PMC 2017 Jun 30. (PMC5493145; doi:10.1016/j.celrep.2017.02.033)
Supplement: Supplemental Experimental Procedures [file NIHMS866722-supplement.pdf]

Cell Reports, Volume *18*

Supplemental Information

Integrative Genomic Analysis of Cholangiocarcinoma Identifies Distinct *IDH*-Mutant Molecular Profiles

Farshad Farshidfar, Siyuan Zheng, Marie-Claude Gingras, Yulia Newton, Juliann Shih, A. Gordon Robertson, Toshinori Hinoue, Katherine A. Hoadley, Ewan A. Gibb, Jason Roszik, Kyle R. Covington, Chia-Chin Wu, Eve Shinbrot, Nicolas Stransky, Apurva Hegde, Ju Dong Yang, Ed Reznik, Sara Sadeghi, Chandra Sekhar Pedamallu, Akinyemi I. Ojesina, Julian M. Hess, J. Todd Auman, Suhn K. Rhie, Reanne Bowlby, Mitesh J. Borad, The Cancer Genome Atlas Network, Andrew X. Zhu, Josh M. Stuart, Chris Sander, Rehan Akbani, Andrew D. Cherniack, Vikram Deshpande, Taofic Mounajjed, Wai Chin Foo, Michael S. Torbenson, David E. Kleiner, Peter W. Laird, David A. Wheeler, Autumn J. McRee, Oliver F. Bathe, Jesper B. Andersen, Nabeel Bardeesy, Lewis R. Roberts, and Lawrence N. Kwong

Integrative Genomic Analysis of Cholangiocarcinoma Identifies Distinct *IDH*-Mutant Molecular Profiles

Inventory of Supplemental Materials

Supplemental Methods

Supplemental Figure 1. Somatic mutations and copy number alterations, related to Figure 1.

Supplemental Figure 2. Initial mRNA clustering of the 38 CCA samples, using the top 400 most-variable genes, related to Figure 2.

Supplemental Figure 3. Characteristics of mitochondrial and chromatin modifier signatures, related to Figure 3.

Supplemental Figure 4. DNA methylation, MicroRNA, lncRNA, and reverse-phase protein array analyses, related to Figure 4.

Supplemental Figure 5. Specific lncRNAs and miR-194-5p correlate with the chromatin modifier signature, related to Figure 5.

Supplemental Figure 6. Three-way cancer comparison analyses among HCC, PDAC, and CCA, related to Figure 6.

Supplemental Table 1. CHOL sample info

Supplemental Table 2. Mutation information for 43 genes that were selected for targeted deep sequencing validation.

Supplemental Table 3. List of fusions identified by multiple algorithms.

Supplemental Table 4. List of 387 liver-specific genes filtered out of the mRNA list prior to PCA.

Supplemental Table 5. Novel genes sets appended to the MSigDb c5 set for GSEA.

Supplemental Table 6. GSEA results identify genes differentially expressed in mRNA Cluster 1 vs 2+3.

Supplemental Table 7. Gene lists for chromatin modifiers and oxidative phosphorylation genes.

Supplemental Table 8. Meta-analysis of published intrahepatic cholangiocarcinoma mutation rates for selected genes.

Supplemental Table 9. Freeze list of cholangiocarcinoma, pancreatic adenocarcinoma, and hepatocellular carcinoma TCGA samples and associated characteristics.

Supplemental Experimental Procedures

Sample Processing

DNA and RNA were extracted, and quality was assessed at the central BCR. RNA and DNA were extracted from tumor and adjacent non-tumor tissue specimens using a modification of the DNA/RNA AllPrep kit (Qiagen). Small RNAs <200 nt were isolated from the Qiagen DNA column flow-through using a *mir*Vana Kit (Ambion) for subsequent miRNA sequencing. DNA was extracted from blood using the QiaAmp DNA Blood Midi kit (Qiagen).

RNA samples were quantified by measuring absorbance at 260nm with an ultraviolet spectrophotometer, and DNA was quantified by PicoGreen assay. To confirm the high molecular weight fragments needed for sequencing, DNA specimens were resolved by 1% agarose gel electrophoresis. A custom Sequenom single-nucleotide polymorphism (SNP) panel or the AmpFISTR Identifiler (Applied Biosystems) was utilized to verify that tumor DNA and germline DNA representing a case were derived from the same patient. 500ng of each tumor and germline DNA were sent to Qiagen (Hilden, Germany) for REPLI-g whole-genome amplification using a 100µg reaction scale. RNA was analyzed via the RNA6000 Nano assay (Agilent), and only analytes with an RNA Integrity Number (RIN)≥7.0, plus a minimum of 6.9 µg of tumor DNA, 5.15 µg of RNA, and 4.9 µg of germline DNA were included in this study.

Sample Qualification

The BCR received tumor samples with germline controls from a total of 84 cases, of which 51 cases qualified and were sent for further genomic analysis. Of the 33 cases that failed to qualify, 2 were disqualified prior to processing, 24 failed for pathology screening, 6 cases failed due to molecular criteria, and 1 failed due to a genotype mismatch between tumor and germline.

Of the 24 that failed pathologic criteria, 21 had insufficient tumor nuclei (<60%), 1 had necrosis (>20%), and 2 had both insufficient tumor nuclei and necrosis. All 6 cases that failed molecular screening had RIN < 7.0 and low molecular weight germline or tumor DNA as measured by gel electrophoresis.

Of the 51 cases that qualified based on BCR pathology review and molecular characteristics, 34 cases were ultimately used for the study set. An additional 4 cases collected for the TCGA hepatocellular carcinoma project (TCGA-FV-A3I0, TCGA-UB-A7MA, TCGA-KR-A7K2, TCGA-BC-A10Q) were determined by the analysis working group to be consistent with CCA and were included in this study set. The cases that were not used in the final analysis were excluded after independent pathology review or discovery of clinical disqualifiers.

Samples with residual tumor tissue after extraction of nucleic acids were considered for proteomics analysis. When available, a 10 to 20 mg piece of snap-frozen tumor adjacent to the piece used for molecular sequencing and characterization was submitted to MD Anderson for reverse-phase protein array (RPPA) analysis.

Histology

A panel of 4 histopathologists with expertise in hepatic pathology evaluated digital slides (via Biopathology Center's Virtual Imaging for Pathology, Education & Research application [VIPER]) for the 38 CCAs and mixed hepatocellular cholangiocarcinomas in this study. Slides consisted of hematoxylin and eosin–stained sections from the formalin-fixed paraffin-embedded tumors and uninvolved hepatic parenchyma scanned at 200x or 400x magnification. Histomorphologic features evaluated included tumor subtype, grade, percent gland formation, infiltration of tumor by lymphocytes and/or neutrophils, degree of fibrosis and necrosis, and the presence or absence of perineural and lymphovascular invasion. Non-neoplastic liver tissue, when available, was evaluated separately for evidence of chronic liver disease. Histomorphologic features evaluated included diagnostic classification; semiquantitative scoring of fibrosis, inflammation, and steatosis; as well as the presence or absence of pseudoxanthomatous change, ductopenia, ductal sclerosis, biliary intraepithelial neoplasia, pigmentation suggestive of iron, and ballooning injury. Consensus among the histopathologists was decided based on majority rule, with ties being decided in favor of lesion presence rather than absence. For scaled observations, the median value (rounding up) was used as the consensus opinion.

DNA Sequencing

Small-insert, dual indexed Illumina paired-end libraries were constructed from 250 ng of genomic DNA on the SciClone NGS instrument (Perkin Elmer, Waltham, MA) with the KAPA HTP sample prep kit according to the manufacturer's recommendations (KAPA Biosystems, Woburn, MA). DNA was fragmented using Covaris (LE220 Covaris, Woburn, MA), targeting a size range of 100–400 bp. Short index sequences (8 bp) were utilized during library construction to enable sample multiplexing. A dual index approach was utilized in which unique sample indices were placed at each end of the ligated library fragment. The final libraries were assessed using the LabChip GX instrument (Perkin Elmer) to validate the concentration and average size (300-500 bp). Libraries for exome capture and validation were pooled and precaptured with slightly different schemas: 1) batch 428 was processed in sets of 9 libraries per pool, receiving 300 ng from each library and 2) libraries for validation were constructed and processed in sets of 23 samples per pool, receiving 217 ng per library.

Exome Capture Method

Each library/pool was hybridized using the Roche NimbleGen SeqCap EZ Human Exome Library v3.0 kit (Roche Nimblegen, Madison, WI) which targets 64 Mb of coding sequence from more than ~20,000 genes. In addition to the exome space, additional space was added through a spike-in procedure using biotinylated 120-bp oligonucleotides (IDT lockdown probes; Integrated DNA Technologies, Coralville, IA) specific for the human papillomavirus promoter region as well as a custom pool of probes targeting hepatitis B viral sequences. These probes were added at a molar equivalent equal to the probe concentration of the exome probes, thus representing those regions in a similar quantity as the exome regions. The library pools were hybridized according to the manufacturer's recommendations with the following exceptions: 1) 5 µg of Human Cot-1 DNA and 1 mM library adapter blockers were added to the hybridization reaction, and 2) each sample was amplified by PCR using 20 µl of enriched single-strand DNA library fragments, KAPA HotStart Polymerase, and 200 nM of each forward and reverse primer.

Validation Capture Method

Each library/pool was hybridized using a Roche NimbleGen SeqCap EZ Choice custom capture reagent (Roche Nimblegen), which targeted ~1 Mb of space. In addition to the custom space, additional probes targeting the telomerase reverse transcriptase (TERT) promoter, hepatitis B virus (HBV) promoter region, and HBV viral sequences (IDT lockdown probes, Integrated DNA Technologies) were included (see

below). The IDT probes were combined in an equal molar equivalent, thus representing those regions in a quantity similar to that of the custom set of NimbleGen probes. The library pools were hybridized according to the manufacturer's recommendations with the following exceptions: 1) 5 µg of Human Cot-1 DNA and 1 mM library adapter blockers were added to the hybridization reaction, and 2) each sample was amplified by PCR using 20 µl of enriched single-strand DNA library fragments, KAPA HotStart Polymerase, and 200 nM of each forward and reverse primer.

Details of the Custom NimbleGen Capture Reagent and IDT Probes

#1. TERT-promoter_-146_-124 on chr5:1295227-1295250.

#2. All somatic mutations for CHOL, excluding mitochondrial sites and overlaps with #3.

#3. All coding exons defined by Gencode 19 (Ensembl 74) in the 48 genes below are targeted.

#4. JC Polyomavirus (NC_001699.1)

#5. Human Herpes virus 1, Herpes Simplex Virus (NC_001806.1), Human Herpes virus 4 type 1, Epstein-Barr Virus (NC_007605.1) and Hepatitis B Virus Genotype G AP007264.1.

Data Generation

The concentration of each capture pool was accurately determined through quantitative PCR according to the manufacturer's protocol (KAPA Biosystems, Inc, Woburn, MA) to produce cluster counts appropriate for the Illumina sequencing platforms.

1) Batch 428. Each capture pool was loaded across two lanes of a HiSeq2000 version 3 flow cell according to the manufacturer's recommendations (Illumina, San Diego, CA). Then 2 X 101bp read pairs were generated for each sample, yielding approximately 6-10 Gb of sequence per sample, which leads to > 80% of the target space covered at 20x with an average mean depth of 50x coverage. Sample identity was confirmed for each sample by comparing sequence data with the TCGA Affymetrix genotype array data.

2) Validation design. Validation was performed with a targeted custom capture that covered each mutated site as well as the complete exonic coding regions of a set of 49 selected genes. Thirty-five of these genes were selected based on their mutation frequency among the TCGA CCA set, their importance as driver genes in HCC, and their general known oncogenic status. The remaining 13 genes were included

in the validation panel to confirm that no mutation could be detected in these genes at deeper coverage. The TERT promoter region was not covered in the original whole-exome sequencing (WES) and was added to the validation panel (Validation list, see below).

**Selection criteria**	**Gene name**			
Cholangiocarcinoma driver genes	ARAF	BRAF	IDH2	PIK3CA
	ARID1A*	FGFR2	KRAS	PTEN*
	BAP1*	IDH1	PBRM1	TP53*
Potential candidates mutated in our panel	ALB*	BRCA2	GNAS	PXMP4
	APC	CDKN2A	KDR	ROR2
	ARID1B	ELF3	MAGEA1	SAV1
	ARID2	ELF4	MLL3/KMT2C	SF3B1
	ATM	EP400	NF2	SMARCD1
	BCL3	ERBB2	NOTCH1	
Not covered in WES discovery	TERT promoter			
Exploratory list	AXIN1*	CDKN1A*	NF1	SMAD4
	CCND1	CTNNB1*	NRAS	
	CCND3	HNF1A*	QKI	
	CDK4	LKB1/STK11	RB1*	

*HCC top 10 smg

WES Analysis

Primary BAM files were analyzed by four calling centers. MAF files from all centers were combined and similarly annotated by checking variant coverage information in the BAM files. Variants were included in the combined final MAF file if called by more than one calling center and validated by a second library/sequencing reaction. BAM files were run separately through Atlas-SNP (Shen et al., 2010), Atlas-Indel, and PInDel (Ye et al., 2009). Data were aggregated for each tumor and normal tissue pair, and variants were cross-checked for each pair. Variant annotation was performed using Annovar (Wang et al., 2010a), COSMIC (Forbes et al., 2011), and dbSNP (Sherry et al., 2001). Variant filtering was performed to remove low-quality variants. Cohort-level data processing was performed to remove additional false somatic calls by filtering against a cohort of normal tissues.

To generate a mutational decomposition for each tumor sample, mutations for this cohort were compared against 21 distinct mutational signatures generated from a set of over 6,000 somatic mutations across a range of cancer types using non-smooth, non-negative matrix factorization (nsNMF) (Pascual-Montano et al., 2006). Samples were aggregated and compared to clinical covariates by using hierarchical clustering

and other correlative statistics. The final MAF file was used to calculate significantly mutated genes using MutSig-CV and an inactivation bias test (Lawrence et al., 2013).

RNA Sequencing

Library Method

From each sample, RNA was extracted, converted into mRNA libraries, sequenced as 50-bp paired-end reads on Illumina HiSeq 2000 Genome Analyzers, and subjected to quality control as previously described (Cancer Genome Atlas Research, 2012). Reads were aligned to the hg19 genome assembly using Mapsplice v12_07 (Wang et al., 2010b). Gene expression was quantified for the transcript models corresponding to the TCGA GAF2.1 (https://tcgadata.nci.nih.gov/docs/GAF/GAF.hg19.June2011.bundle/outputs/TCGA.hg19.June2011.gaf), using RSEM (Li and Dewey, 2011) and normalized within a sample to a fixed upper quartile. For further details on this processing, refer to the description file at the Data Computing Center data portal under the V2_MapSpliceRSEM workflow (https://tcga-data.nci.nih.gov/tcgafiles/ftp_auth/distro_ftpusers/anonymous/tumor/cesc/cgcc/unc.edu/illuminahiseq_rnaseqv2/rnaseqv2/unc.edu_CESC.IlluminaHiSeq_RNASeqV2.mage-tab.1.9.0/DESCRIPTION.txt).

Unsupervised Expression Clustering

The normalized RSEM values for the tumor samples were $\log_2$ transformed after adding a constant of “1” to all values. The gene expression matrix was further filtered to include only the top 10% most variable genes by mean absolute deviation (n=2,053 genes). Consensus clustering using self-organized maps was employed to identify the most robust expression clusters for 2 to 6 clusters. Rank survey profiles for the cophenetic and silhouette widths, along with consensus cluster membership heatmaps (data not shown) suggested that a 2-cluster solution was optimal. A nearest centroid-based classifier (CLaNC) (Dabney, 2006) was used to identify a set of 400 signature genes that had the lowest cross-validation and prediction errors for sample membership in their respective clusters. Hierarchical clustering was performed after median centering gene expression values using Cluster 3.0 (de Hoon et al., 2004) (uncentered correlation with centroid linkage) and was visualized using Java TreeView (Saldanha, 2004).

Data Filtering

Of the 20,531 transcripts identified on RNA-seq, 15,272 transcripts were expressed in more than 80% of cases. Genes highly expressed in liver parenchyma (541 genes) were subtracted. Expression values greater than 4 standard deviations from the mean were replaced with a value corresponding to 2

standard deviations from the mean, to impute outliers. The expression values for the remaining 14,911 genes were log transformed and auto-scaled. Principal component analysis (PCA) and orthogonal partial least squares discriminant analysis (OPLS-DA) were performed using SIMCA Version 14 (Umetrics AB, Sweden). PCA was applied to explore intrinsic patterns of gene expression. Expression data variation was projected into 7 major principal components, and each sample's component score was then calculated. The resulting PCA scores were used as distance metrics for hierarchical clustering, based on the method of Ward (Ward, 1963).

To identify genes that contributed most to the cluster separation, a supervised multivariate discriminant analysis OPLS-DA was performed (Bylesjö et al., 2006). The most significant genes were selected by variable importance in projection. Three times we trimmed out genes with variable importance in projection score lower than average, resulting in a list of 1,150 genes. The generated model was assessed for goodness of fit ($0 \le R2Y < 1$), goodness of predictability ($0 \le Q2Y \le 1$), and cross validation reliability P value (CV-ANOVA). To demonstrate reproducibility of the model's predictions, the permutation test on sample labeling was applied 999 times to evaluate the separation of each cluster combination. In each case, the Q2Y intercept was substantially below zero, which implies that the gene set contained in the model is robust and highly reproducible. Inter-gene correlations and clustering were evaluated by hierarchical clustering of the 1,150 genes comprising the transcriptional model. Complete linkage on Pearson correlation distance metrics was used for hierarchical clustering. A heatmap, representing gene clusters for each cluster group, was constructed using Multiexperiment Viewer (MeV) (Saeed et al., 2003). The transcriptional clustering identified using TCGA RNA-seq data was validated by comparison to an external dataset (Andersen et al., 2012). In the external data set, a total of 976 genes of the RNA-seq transcripts overlapped. Outliers in the external validation cohort were detected using PCA on log-transformed and auto-scaled expression data. Two samples were excluded because their transcriptional profile was situated outside of the 95% confidence interval. The remaining samples were subjected to median fold change normalization to account for inter-sample differences. To enable comparison between the two different platforms, we applied a surrogate variable analysis (SVA) method, the ComBat method(Leek et al., 2012), which adjusted the external dataset to reside in the same scale as the TCGA RNA-seq data. The external dataset was compared to the TCGA data set by means of sample-wise hierarchical clustering on the 976 genes shared between datasets. For the sake of comparison,

gene orders were kept the same as the order generated by MeV in the TCGA dataset. Functional pathways affected were evaluated using Ingenuity Pathway Analysis (IPA) on the genes that were differentially upregulated in the transcriptional cluster containing *IDH1* mutations.

Identification of Long Noncoding RNAs

RNA-seq files were downloaded from CGHub, and sequence reads were aligned to the human reference genome (hg38) and transcriptome (Ensembl v.82 gene models) using STAR version 2.4.2a. STAR was run with the following parameters: minimum/maximum intron size of 30 and 500,000, respectively; noncanonical, unannotated junctions were removed; maximum tolerated mismatches was set to 10; and the outSAMstrandField intron motif option was enabled. The Cuffquant command included with Cufflinks v.2.2.1 (Trapnell et al., 2010) was used to quantify the read abundances per sample, with fragment bias correction and multiread correction enabled, and all other options set to default. To calculate the fragments per kilobase of exons per million fragments mapped (FPKM), the Cuffnorm command was used with default parameters. From the FPKM matrix for 38 tumors and 11 adjacent normal samples, we extracted genes with “lincRNA” and “processed_transcript” biotypes.

Unsupervised Clustering of lncRNAs

For 38 tumor and 11 adjacent normal samples we extracted 344 highly variable and robustly expressed lncRNAs (FPKM variance above the 95th percentile and mean FPKM ≥1) from a normalized abundance matrix of 8167 Ensembl v82 lncRNAs (i.e., lincRNA and processed transcripts). We applied unsupervised consensus clustering to the matrix with ConsensusClusterPlus v1.24.0 in R 3.2.1 (Wilkerson and Hayes, 2010), generating clustering solutions for between 2 and 7 clusters for Spearman and Pearson hierarchical, PAM and k-means runs. Overall, from consensus membership results, runs using Spearman correlations, k-means clustering on distance matrices (kmdist), and 10,000 iterations gave the best solutions. After assessing consensus heatmaps, dendrograms, and distribution functions; Kaplan-Meier survival plots; and covariate tracks representing clinical parameters and clustering results from other platforms, we reported a four-cluster solution. To generate a heatmap, we selected a subset of lncRNAs that was highly scored in a significance analysis of microarrays (SAM) multiclass analysis, transformed each row of the matrix by $\log_{10}$(FPKM + 1), then used the pheatmap R package (v1.0.2) to scale and cluster only the rows, using a Euclidean distance metric and WardD2 clustering.

Expression Comparison across Tumor Types

A data matrix of normalized gene-level RSEM values from 38 CCA (CHOL), 196 TCGA liver hepatocellular carcinoma (LIHC), and 151 TCGA pancreatic adenocarcinoma (PAAD) samples was used to identify expression patterns across the 3 cancer types. The normalized RSEM values for the data matrix were $\log_2$ transformed, and genes with >10% missing values across samples were removed. The gene expression matrix was further filtered to include only the top 10% most variable genes by mean absolute deviation (n=1,595 genes). Hierarchical clustering was performed after median centering of the gene expression values, and after hierarchical clustering the expression patterns were visualized using Java TreeView.

Analysis of Fusion Events

The deFuse software, version 0.6.1 (McPherson et al., 2011) with default settings, was used to detect fusion genes. A list of candidate fusion genes was generated by further filtering out identified read-through fusions, selecting coding regions, selecting in-frame open reading frame (ORF) genes, and selecting samples with a deFuse confidence score of >80%. To remove genes that mapped to multiple locations, each spanning-junction read of the candidate fusion genes was examined using the BLAT tool in the UCSC genome browser. The fusions that mapped with 100% identity to each part of the identified fusion (gene1 or gene2) were selected for further analysis. Next, each RNA BAM from candidate fusion genes was examined in IGV (Integrative Genomics Viewer, Broad Institute) (Robinson et al., 2011; Thorvaldsdottir et al., 2013) by looking for stacked unaligned sequence and changes in coverage at the identified fusion break points. Each unaligned sequence was brought into the UCSC genome browser and mapped using BLAT. Only fusions that had reads that matched (100%) the identified fusion genes were considered further.

Integrative analysis of multiple fusion-detection methods has been shown to significantly reduce false-positive/negative calling of fusion genes. In addition to the deFuse software (McPherson et al., 2011), we utilized four other algorithms to predict fusion genes, including TopHat-Fusion (Kim and Salzberg, 2011), MapSplice (Wang et al., 2010b), FusionMap (Ge et al., 2011), and PRADA (Torres-Garcia et al., 2014). For all tools, we selected only fusions with one minimum junction read and two supporting mate pairs that

mapped to candidate gene pairs. A total of 5,107 fusions was detected by at least one of the tools, but only 118 were detected by at least two tools. By incorporating copy number data, we found that 47.54% of the 118 fusions detected by at least two tools had significant intragenic copy number changes in at least one of the parental genes. Finally, we applied a fusion centrality approach (Wu et al., 2013) to prioritize potential drivers from the predicted 118 fusion events. In total, we identified 48 potential fusion drivers in 26 of CCA samples, consisting of 10 inter-chromosomal and 38 intra-chromosomal fusions with a total of 23 in-frame fusions. *FGFR2* fusions were detected in 5 samples, including FGFR2-BICC1 (n=2), FGFR2-KIAA1598 (n=1), FGFR2-FRK (n=1), and FGFR2-C10ORF118 (n=1). Other fusion genes detected include *NRAS, ERBB2, ITGAV, CRK, TAOK1, MAPKAPK5, CCL14*, and *NF1*.

MicroRNA Sequencing

Data Generation and Preprocessing

MicroRNA sequence (miRNA-seq) data were generated using methods described previously (Cancer Genome Atlas, 2012; Chu et al., 2016) except that 1 ug of total RNA (at 250 ng/uL) was used as input instead of messenger RNA-depleted RNA. We aligned reads to the GRCh37/hg19 reference human genome and annotated miRNAs with miRBase v16, using only exact-match read alignments. miRNA-seq BAM files that are available from cgHUB (cghub.ucsc.edu) (Wilks et al., 2014) include all sequence reads. We used miRBase v20 to assign 5p and 3p mature strand names to MIMAT accession IDs.

Unsupervised Clustering of miRNA Mature Strands

For the 38 tumor samples, we used unsupervised non-negative matrix factorization (NMF) consensus clustering (v0.20.5) in R 3.1.2, with default settings (Gaujoux and Seoighe, 2010). The input was a reads-per-million (RPM) data matrix for the 303 (25% of 1,212) miRBase v16 5p or 3p mature strands (i.e., miRs) that had the largest variances across the cohort. After running a rank survey with 50 iterations per solution, we chose a clustering solution (i.e., number of clusters) by evaluating profiles of the cophenetic correlation coefficient and the average silhouette width calculated from the consensus membership matrix, Kaplan-Meier plots, and clinical covariate associations, then performed a 500-iteration final clustering run. To visualize typical vs. atypical cluster members, we calculated a profile of silhouette widths from the final NMF consensus membership matrix. To generate a heatmap we first identified miRNAs that were differentially abundant across the unsupervised miRNA clusters and 9 adjacent normal

tissues by using a SAM multiclass analysis (samr 2.0) (Li and Tibshirani, 2013) in R 3.2.1, with an RPM input matrix and a false-discovery rate (FDR) threshold of 0.05. We displayed miRNAs that had high SAM scores and median abundances >25 RPM; the RPM filtering acknowledged that miRNAs that are more abundant are more likely to be influential (Mullokandov et al., 2012; Tay et al., 2014). We transformed each row of the matrix by $\log_{10}$(RPM + 1), then used the pheatmap v1.0.2 R package to scale and cluster only the rows, with a Euclidean distance metric and Ward clustering.

To determine differentially abundant miRNAs and genes, we identified miRNAs and genes that were differentially abundant by using unpaired two-class and multiclass SAM analyses (samr v2.0), with an RPM or FPKM input matrix, respectively, and an FDR threshold of 0.05. For clinical and molecular covariates, we calculated contingency table association *P* values using R, with a chi-square or Fisher exact test for categorical data, and a Kruskal-Wallis test for real-valued data. Tumor sample purity and ploidy were calculated using ABSOLUTE (Carter et al., 2012).

We assessed potential miRNA-gene targeting for all tumor samples by calculating miRNA-mRNA Spearman correlations with MatrixEQTL v2.1.1, using gene-level normalized abundance RNA-seq (RSEM) data. We calculated correlations with a *P* value threshold of 0.05, then filtered the anticorrelations at FDR<0.05. We extracted miRNA-gene pairs that corresponded to functional validation publications reported by miRTarBase v6.0(Hsu et al., 2014). The results were displayed with Cytoscape v2.8.3.

SNP Array-based Copy Number Analysis

Somatic copy number data were generated on Affymetrix SNP 6.0 arrays using standard protocols at the Genome Analysis Platform of the Broad Institute (McCarroll et al., 2008). Briefly, preliminary copy number at each probe locus was inferred by Birdseed analysis of raw .CEL files (Korn et al., 2008).). Tangent normalization was then used to further refine genome wide copy number estimates (http://www.broadinstitute.org/cancer/cga/copynumber_pipeline). From this, segmented copy number data were generated using Circular Binary Segmentation (Olshen et al., 2004). Regions corresponding to germline copy number alterations were removed by applying filters generated from normal samples. Regions of significantly reoccurring somatic copy number alterations were identified using GISTIC 2.0

analysis (Mermel et al., 2011). Allelic copy number, whole-genome doubling, subclonality, and purity and ploidy estimates were calculated using ABSOLUTE. For samples with ABSOLUTE corrected copy number, Circular Binary Segmentation (CBS)-derived segmented copy number values were recentered using the In Silico Admixture Removal (ISAR) procedure (Zack et al., 2013). Significant focal copy number alterations were identified from ISAR-corrected segmented data using GISTIC 2.0.225. For copy number–based clustering, tumors were clustered based on threshold copy number variation at recurring alteration peaks from GISTIC analysis (all_lesions.conf_99.txt file). Clustering was done in R based on Manhattan distance using Ward's method.

DNA Methylation

The Illumina Infinium DNA methylation platform HumanMethylation450K (Illumina, San Diego, CA) was used to obtain DNA methylation profiles of CHOL samples. The DNA methylation score is represented as a beta (β) value β = [M/(M+U)] in which M and U indicate the mean methylated and unmethylated signal intensities, respectively. β values range from 0 to 1, with scores of 0 indicating no DNA methylation and scores of 1 indicating complete DNA methylation. A detection *P* value accompanies each data point and compares the signal intensity difference between the analytical probes and a set of negative control probes on the array. Any data point with a *P* value > 0.05 is deemed not to be statistically significantly different from background and is thus masked as “NA” in the level 3 data packages, as described below.

Sample and Data Processing

Bisulfite conversion was performed on 1 µg of genomic DNA from each sample using the EZ-96 DNA Methylation Kit (Zymo Research, Irvine, CA) according to the manufacturer’s instructions. The amount of bisulfite-converted DNA and completeness of bisulfite conversion was assessed using a panel of MethyLight-based quality control (QC) reactions (Campan et al., 2009). All TCGA samples passed our QC tests and were then whole-genome amplified (WGA) and enzymatically fragmented. Samples were then hybridized to Infinium DNA methylation arrays and these were scanned using Illumina iScan. Raw IDAT files were processed using R/Bioconductor package methylumi. TCGA DNA methylation data packages were then generated using the EGC.tools R package (https://github.com/uscepigenomecenter/EGC.tools). Data are available at TCGA Data Portal website

(http://tcga-data.nci.nih.gov/tcga/). A disease-mapping file (CHOL.mappings.csv) is provided in the AUX directory to facilitate this process.

Level 2 data contain background-corrected methylated (M) and unmethylated (U) summary intensities as extracted by methylumi. Nondetection probabilities (*P* values) were computed as the minimum of the two values (one per allele) for the empirical cumulative density function of the negative control probes in the appropriate color channel. Background correction was performed via normal-exponential deconvolution. Multiple-batch archives had the intensities in each of the two channels multiplicatively scaled to match a reference sample (sample with reg/green ratio of the normalization control probes closest to 1.0).

Level 3 data contain β-value calculations with annotations for HUGO Gene Nomenclature Committee (HGNC) gene symbol, chromosome, and genomic coordinates (UCSC hg19, Feb 2009) for each targeted CpG/CpH site on the array. Probes having a common SNP (minor allele frequency > 0.01, per dbSNP build 135 via the UCSC snp135common track) within 10 bp of the interrogated CpG site or having a 15-bp distance from the interrogated CpG site which overlapped with a repetitive element (as defined by RepeatMasker and Tandem Repeat Finder Masks contained in the BSgenome.Hsapiens.UCSC.hg19 R package), were masked as "NA" across all samples, and probes with a nondetection probability (*P* value) > 0.05 in a given sample were masked as "NA" on that array. Probes that were mapped to multiple sites on hg19 were annotated as "NA" for chromosome and 0 for CpG/CpH coordinate. Data from the following archives were used for the analyses described in this manuscript.

1) jhu-usc.edu_CHOL.HumanMethylation450.Level_3.1.1.0
2) jhu-usc.edu_LIHC.HumanMethylation450.Level_3.1.13.0
3) jhu-usc.edu_LIHC.HumanMethylation450.Level_3.6.13.0
4) jhu-usc.edu_LIHC.HumanMethylation450.Level_3.13.13.0

Unsupervised Clustering Analysis of DNA Methylation Data

Level 3 DNA methylation data contained in the packages listed above were used for analysis. Probes that had any "NA"-masked data points or probes designed for sequences on X and Y chromosomes were removed.

To capture cancer-specific DNA hypermethylation events, we first selected CpG sites that were not methylated in normal tissues (mean β value <0.2). To minimize the potential influence of variable levels of tumor purity among CHOL samples on the clustering result, CpG sites that are methylated in leukocytes were removed (mean β value >0.2). We then dichotomized the data using a β value of >0.3 as a threshold for positive DNA methylation. The dichotomization not only ameliorated the effect of tumor sample purity on the clustering, but also removed a great portion of residual batch or platform effects that are mostly reflected in small variations near the two ends of the range of β values. Next, unsupervised hierarchical clustering was performed on 37,743 CpG sites that were methylated, with this threshold in at least 10% of the tumors using a binary distance metric for clustering and Ward's method for linkage. The heatmap was generated based on the original β values, visualizing a subset (25%) of 9,434 CpG sites.

To determine genes silenced by DNA methylation, we first removed DNA methylation probes overlapping with SNPs, repeats, or those designed for sequences on X and Y chromosomes and non-CpG sites. The remaining probes were mapped against UCSC Genes using the GenomicFeatures R/Bioconductor package. Probes that were located in a promoter region (defined as the 3-kb region spanning from 1,500 bp upstream to 1,500 bp downstream of the transcription start site) were identified. Level 3 RNA-seq reads per kilobase of transcript per million mapped reads (RPKM) data were $\log_2$ transformed [$\log_2$ (RPKM+1)] and used to assess the expression levels associated with DNA methylation changes. DNA methylation and gene expression data were merged by Entrez Gene IDs. We removed the CpG sites that were methylated in normal tissues (mean β value >0.3). We then dichotomized the DNA methylation data using a β value of >0.3 as a threshold for positive DNA methylation and further eliminated CpG sites methylated in <5% of the tumor samples. For each probe-gene pair, we applied the following algorithm: 1) organize the tumors as either methylated (β≥0.3) or unmethylated (β<0.3); 2) compute the mean expression in the methylated and unmethylated groups; and 3) compute the standard deviation of the expression in the unmethylated group. We then selected probes for which the mean expression in the methylated group was lower than 1.64 standard deviations of the mean expression in the unmethylated group. We labeled each tumor sample as epigenetically silenced for a specific probe-gene pair selected from above if 1) it belonged to the methylated group and 2) the expression of the corresponding gene was lower than the mean of the unmethylated group of samples. If multiple probes were associated with the same gene, a sample identified as epigenetically silenced at more than half the probes for the

corresponding gene was also labeled as epigenetically silenced at the gene level. Statistical analysis and data visualization were carried out using the R/Biocoductor software packages (http://www.bioconductor.org).

Pathogen Detection from RNA-seq Data

The PathSeq algorithm (Kostic et al., 2011) was used to perform computational subtraction of human reads, followed by alignment of residual reads to a combined database of human reference genomes and microbial reference genomes (which includes but is not limited to HBV genomes), resulting in the identification of reads mapping to HBV genomes in RNA-seq data. Subjects were classified as HBV positive by RNA-seq if at least 1 HBV read in 1 million human reads was present; otherwise, subjects were classified as HBV negative. Using PathSeq, human reads were subtracted by first mapping reads to a database of human genomes using Burrows-Wheeler Aligner (BWA) (version 0.6.1) (Li and Durbin, 2009), Megablast (version 2.2.23), and Blastn (version 2.2.23) (Altschul et al., 1997). Only sequences with perfect or near-perfect matches to the human genome were removed in the subtraction process. To identify HBV reads, the resultant nonhuman reads were aligned with Megablast to a database of microbial genomes that includes bacteria, archaea, viruses, and fungi reference genomes (downloaded June 2012).

Identification of HBV Integration Sites

An HBV-positive sample was considered integration positive if there were at least 3 spanning reads and 10 flanking reads supporting an integration event. Flanking read pairs were defined as having one end of the paired-end read mapped to the HBV genome and its mate pair mapped to the human genome. Spanning reads were defined as having one end of the paired-end read spanning the integration junction and its mate pair mapped to either the human or HBV genome. Once HBV reads were obtained, we extracted all pair mates and used TopHat 2.0.8 (Kim et al., 2013) with the fusion option enabled to map these paired-end reads to a combined database containing the human genome and an HBV genome. Next, spanning reads and flanking reads were identified from the aligned BAM file. Human genes involved in the integration were identified using the breakpoint coordinates against RefSeq and UCSC gene annotations (last modified on 30-Jun-2013) from the UCSC genome browser.

Microbial Detection

This pipeline is based on BBT (Release 1.2.10) (Chu et al., 2014), a Bloom filter–based method for rapid sequence classification of sequenced reads. BBT was run with a sliding window size (i.e., k-mer length) of 25 bp and an FDR of 0.02. We generated 43 filters used in this analysis from complete NCBI genome reference sequences for microbial species that included bacteria, viruses, fungi, and protozoa. In a single-pass scan, BBT categorizes each read as matching the human or a single, specific microbe, as matching two or more species (multimatch), or as matching none of the filters (no match). For each filter, we calculated a reads per million mapped reads (RPM) abundance metric and applied a threshold of 0.2 RPM to identify samples as being positive for microbial presence.

$$Abundance\ metric = \left(\frac{\#reads\ mapped\ to\ the\ microbe}{\#reads\ mapped\ to\ human\ in\ the\ sample} * 10^6 \right)$$

For cases with RPMs that were near or above the 0.2 RPM screening threshold, we assessed whether viruses had integrated into the human genome. For WES libraries from the 38 CCAs, we assessed human herpes virus in 25 libraries (HHV4 in 3X-AAV9, 3X-AAVB, 4G-AAZT, W5-AA2G, W5-AA2H, W5-AA2R, W5-AA31, WD-A7RX, ZD-A8I3 and HHV6A in W5-AA2R, W5-AA31) and assessed hepatitis A, B, or C in 2 exome libraries (3X-AAVE). For RNA-seq libraries, we assessed HHV4 in W5-AA2H, and hepatitis A, B, or C in 3X-AAVE. We performed *de novo* assembly (Robertson et al., 2010) with ABySS v1.3.4 using every fourth k-mer value from k=52-96 for WES data and from k=24-48 for RNA-seq data, on each library. For human herpes virus analysis, only the reads classified by BBT as human, human herpes virus, multimatch, and no match were used in the assemblies. For hepatitis analysis, only the reads classified by BBT as human, hepatitis, multimatch, or no match were used in the assemblies. The k-mer assemblies for each library were then merged with Trans-ABySS v1.4.8 to generate the working contig set. BBT was re-run on these contigs, applying only human and either herpes virus or hepatitis filters, to identify contigs that matched both filters. The integration breakpoint in these multimatched contigs was identified by aligning each contig to human GRCh37/hg19 reference sequence and to 109 herpes virus reference sequences or 9 hepatitis reference sequences using BLAT v34 (Kent, 2002). We retained contig alignments in which a) the aligned human and viral sequences summed to at least 90% of the contig length and b) the human and viral alignment overlapped by less than 50%. Human breakpoint coordinates were annotated against RefSeq and UCSC gene annotations (downloaded from the UCSC

genome browser on 30-Jun-2013) (Kuhn et al., 2013). Breakpoints that had at least 3 spanning mate-pair reads or 5 flanking mate-pair reads were considered potential integration sites.

Tumor Map

The Tumor Map represents a dimensionality reduction and visualization method for high-dimensional genomic data. It allows viewing and browsing relationships between high-dimensional heterogeneous genomic samples in a 2-dimensional map, analogous to exploring geographical maps in the Google Maps web application. Samples are arranged in a 2-dimensional space and then associated to hexagons in a regular hexagonal grid. The relative distances in the map approximate the relative similarities between the samples in the original high-dimensional genomic space. Samples with similar genomic profiles are placed near each other in the map. Samples that are less similar are farther away from each other. Given that such relations are preserved, clusters of samples that appear as “islands” in the map will indicate groups of samples that share genomic and/or epigenomic events.

Briefly, Tumor Map utilizes nearest neighborhoods and pair-wise Pearson correlations to project high-dimensional genomic data onto a 2-dimensional grid (see Supplemental Methods), laying out samples to form a landscape-like topology representing sample relationships, similar to an earlier mapping program (Stuart et al., 2003). Tumor Map uses established algorithms, including VxOrd (Martin et al., 2011) to lay out samples and Spring Embedded Layout (Ceccarelli et al., 2016) to cluster graph structures.

To build a tri-cancer multiplatform map, we combined tumor mRNA expression, copy number variation, and methylation profiles for CCA (CHOL), liver hepatocellular carcinoma (LIHC), and pancreatic adenocarcinoma (PAAD). First, we combined mRNA expression data from RNA-seq for CHOL, LIHC, and PAAD into a single dataset. Second, we combined copy number GISTIC calls into a single dataset. Third, we combined methylation profiles from the HumanMethylation450 platform into a single dataset. We computed sample-by-sample pair-wise similarities for each dataset, producing 3 square similarity matrices. We used Spearman rank correlation as a similarity measure on these continuous-valued datasets (mRNA expression, copy number variation, and methylation). Next, we standardized each similarity matrix using the Context Likelihood of Relatedness (CLR) approach (Faith et al., 2007). Given a similarity matrix as input, this method outputs a set of relative similarities, where each similarity pair

reflects how similar the two samples are to each other compared to how similar each is to any sample in a particular cohort. More formally, we compute the standardized similarity measure $Z_D(i,j)$ between two samples i and j from the dth dataset:

$$Z_d(i,j) = \frac{1}{2}\left(\frac{S_d(i,j) - m_d(i)}{\sqrt{v_d(i)}} + \frac{S_d(i,j) - m_d(j)}{\sqrt{v_d(j)}} \right),$$

where $m_d(k)$ is the mean and $v_d(k)$ is the variance of the similarities to a particular sample k in dataset $\underline{d}$. We compute integrated similarity measures Z^* from the relative Z-scores across D different data platforms as:

$$Z^*(i,j) = \frac{\sum_{d=1}^{D} I(d,i) \cdot I(d,j) \cdot Z_d(i,j)}{\sum_{d=1}^{D} I(d,i) \cdot I(d,j)},$$

where $I(d,k)$ records whether sample k has data in dataset d. Thus, Z* represents a simple averaging of the relative similarities between two samples across only those datasets for which both of the samples have valid (non-missing) observations. We note that Z* could incorporate weightings for each dataset w_d indicating the importance of each of the D platforms being combined. However, we did not explore this option for the analysis presented here. The resulting standardized similarity matrix is a square samples-by-samples matrix where the samples are the union of all samples represented in the platforms.

To build the map layout, the closest neighborhood of 10 samples was selected for each sample from the standardized integrated similarity matrix. We represent the local neighborhoods as a graph where the nodes are the samples, and an edge links any two samples if one of them is in the top 10 neighbors of the other. The magnitude of the similarity was used as the edge weight. An X-Y position in the 2-dimensional plane was calculated from the graph using a spring-embedded graph layout algorithm implemented in the Distributed Recursive Graph Layout toolbox (https://github.com/SciTechStrategies/OpenOrd). The spring-embedded layout algorithm treats edges as springs and allows the springs to oscillate for a fixed amount of time with energy that is inversely proportional to the edge weights. Under these conditions, springs with large weights do not oscillate much, causing those vertices to stay together. However, springs with small weights oscillate more and end up farther away from each other. This method enables us to construct a 2-dimensional spatial layout

of the graph with clusters of samples forming clique-like hub sub-structures. Our method then associates each of the nodes with a fixed location on a 2-dimensional hexagonal grid. Each hexagon-shaped cell in the grid can be assigned no more than one vertex, and some can be assigned none representing “empty space” in the map. If multiple vertices compete for the same grid cell, a random vertex selection is made and placed into the cell, and the other competing vertices are assigned to neighboring empty cells using a greedy strategy, snapping around the original cell in a spiral-like manner.

Tumor Map–based Clustering of mRNA Expression Data

We utilized the Tumor Map method to perform unsupervised joint PAAD, LIHC, and CHOL mRNA cluster analysis. We combined mRNA expression RNA-seq data for the 3 cancers and excluded all liver-specific genes from the feature space. We analyzed the remaining 15,269 genes for 588 samples. We computed Tumor Map Euclidean space (x,y) coordinates for each sample, using 6 closest neighbors to render the map. We then computed Euclidean distance between each pair of samples based on the (x,y) coordinates and performed k-means clustering based on those distances. We chose 7 clusters as the best solution because it best recapitulated the expected biological relationships between the tumors (Supplemental Fig. 11 E,F).

Cluster-of-Clusters Analysis (COCA)

We performed cluster-of-clusters analysis (COCA) to obtain an overview of the CCA molecular subtypes for 38 cases with overlapping copy number, miRNA, mRNA, and methylation data. Reverse-phase protein array (RPPA)-derived subtypes were not included. We used the R package “CLUE” to perform COCA. The membership assignment in a cluster solution was dichotomized into 0 and 1 and subsequently converted into a matrix that consisted of all clusters. We assigned equal weight to each cluster solution in COCA. We tested k from 2 to 5. We used a fixed-point algorithm “GV1” and 2,000 iterations. Fisher’s exact test was used to calculate enrichment of molecular and histological features in COCAs.

Pan-cancer Mitchondrial Correlation

Spearman correlation coefficients of gene expressions were calculated using the ‘Hmisc’ R package for the following two sets of genes—chromatin modifiers and those involved in mitochondria structure and

function (see Supplemental Table 10 for a list of genes). The correlation coefficients of CCA and 25 other cancers were visualized using the Tableau Desktop software (http://www.tableau.com/products/desktop). Only correlations with $P < 0.05$ were included in Figure 4F.

RPPA for CHOL-LIHC-PAAD

Protein was extracted using RPPA lysis buffer (1% Triton X-100, 50 mmol/L HEPES [pH 7.4], 150 mmol/L NaCl, 1.5 mmol/L $MgCl_2$, 1 mmol/L EGTA, 100 mmol/L NaF, 10 mmol/L NaPPi, 10% glycerol, 1 mmol/L phenylmethylsulfonyl fluoride, 1 mmol/L Na_3VO_4, and aprotinin 10 ug/mL) from human tumors, and RPPA was performed as previously described (Hennessy et al., 2007; Hu et al., 2007; Liang et al., 2007; Tibes et al., 2006). Lysis buffer was used to lyse frozen tumors by Precellys homogenization. Tumor lysates were adjusted to 1 µg/µL concentration as assessed by bicinchoninic acid assay and boiled with 1% sodium dodecyl sulfate (SDS). Tumor lysates were manually serially diluted two-fold through 5 dilutions with lysis buffer. An Aushon Biosystems 2470 arrayer (Burlington, MA) printed 1,056 samples on nitrocellulose-coated slides (Grace Bio-Labs). Slides were probed with 218 validated primary antibodies followed by corresponding secondary antibodies (goat anti-rabbit IgG, goat anti-mouse IgG, or rabbit anti-goat IgG). Signal was captured using a DakoCytomation-catalyzed system and diaminobenzidene (DAB) colorimetric reaction. Slides were scanned in a CanoScan 9000F. Spot intensities were analyzed and quantified using an Array-Pro Analyzer (Media Cybernetics, Washington, DC) to generate spot signal intensities (level 1 data). The software SuperCurveGUI (Hu et al., 2007) available at http://bioinformatics.mdanderson.org/Software/supercurve/) was used to estimate the EC_{50} values of the proteins in each dilution series (in $\log_2$ scale). Briefly, the nonparametric, monotone increasing B-spline model (Tibes et al., 2006) was used to plot a fitted curve with the signal intensities on the Y-axis and the relative $\log_2$ concentration of each protein on the X-axis using. The raw spot intensity data were adjusted to correct for spatial bias before model fitting. A QC metric was returned for each slide to help determine the quality of the slide: if the score was < 0.8 on a scale of 0-1, the slide was eliminated. The staining was repeated to obtain a high-quality score for most cases. The slide with the highest QC score for each sample was used for analysis (level 2 data). Protein measurements were corrected for loading using median centering across antibodies (level 3 data) (Gonzalez-Angulo et al., 2011; Hennessy et al., 2010). In total, 223 samples (30 CCA, 47 PDAC, and 146 HCC) and 197 antibodies were used. Final selection of antibodies was restricted by the availability of high-quality antibodies that consistently pass a strict

validation process which includes assaying for high specificity, quantification, and sensitivity (dynamic range) using protein extracts from cultured cells or tumor tissue (Hennessy et al., 2007).

RPPA arrays were quantitated and processed (including normalization and load controlling) as previously described using MicroVigene (VigeneTech, Inc., Carlisle, MA) and the R package SuperCurve (version 1.3), available at http://bioinformatics.mdanderson.org/OOMPA (Hu et al., 2007; Tibes et al., 2006). Raw data (level 1), SuperCurve nonparameteric model fitting on a single array (level 2), and loading-corrected data (level 3) were deposited at the Data Coordinating Center.

To correct for sample loading differences, the RPPA data were subjected to median centering across all antibodies for each sample. Natural variations in protein concentrations per unit volume account for the loading differences, due to several factors such as differences in protein concentrations of large and small cells, differences in the amount of protein per cell, or heterogeneity of the cells comprising the samples. Thus, loading differences can be estimated by the expression levels across many different proteins in a sample compared with other samples. To compare protein expression across samples, we subtracted the median protein expression levels, forcing the median value to become zero. These data were used for the analysis of CHOL samples.

However, the analysis of CHOL, PAAD, and LIHC samples together presented the potential problem of batch effects when trying to merge them together, since these were run in different batches. To address this problem, we used Replicates Based Normalization (RBN), an algorithm we developed that uses replicate samples run across multiple batches to adjust the data for batch effects. The underlying hypothesis is that any observed variation between replicates in different batches is primarily due to linear batch effects plus a component due to random noise. Given a sufficiently large number of replicates, the random noise is expected to cancel out since it has a mean of zero, by definition. Remaining differences are treated as systemic batch effects. We can compute those effects for each antibody and subtract them out from the slide.

GTEx determination of immune and liver-specific lncRNAs

The following criteria were applied to identify tissue-specific lncRNAs from an initial list of 7267: 1) the lncRNAs had to be expressed in the tissue of interest at least 10-fold higher than the median of all 52 tissues and 2) the absolute RPKM of the gene in the tissue of interest had to be at least 0.25. The cutoff of 0.25 RPKM was determined through an analysis of genes that passed criteria 1: below this cutoff, the majority of expression in other tissues was zero, which meant that the expression of the gene could not be reliably differentiated from random noise.

Supplemental Methods Reference List

Altschul, S. F., Madden, T. L., Schaffer, A. A., Zhang, J., Zhang, Z., Miller, W., and Lipman, D. J. (1997). Gapped BLAST and PSI-BLAST: a new generation of protein database search programs. Nucleic Acids Res *25*, 3389-3402.

Andersen, J. B., Spee, B., Blechacz, B. R., Avital, I., Komuta, M., Barbour, A., Conner, E. A., Gillen, M. C., Roskams, T., Roberts, L. R.*, et al.* (2012). Genomic and genetic characterization of cholangiocarcinoma identifies therapeutic targets for tyrosine kinase inhibitors. Gastroenterology *142*, 1021-1031 e1015.

Bylesjö, M., Rantalainen, M., Cloarec, O., Nicholson, J. K., Holmes, E., and Trygg, J. (2006). OPLS discriminant analysis: combining the strengths of PLS-DA and SIMCA classification. Journal of Chemometrics *20*, 341-351.

Campan, M., Weisenberger, D. J., Trinh, B., and Laird, P. W. (2009). MethyLight. Methods in molecular biology *507*, 325-337.

Cancer Genome Atlas, N. (2012). Comprehensive molecular portraits of human breast tumours. Nature *490*, 61-70.

Cancer Genome Atlas Research, N. (2011). Integrated genomic analyses of ovarian carcinoma. Nature *474*, 609-615.

Cancer Genome Atlas Research, N. (2012). Comprehensive genomic characterization of squamous cell lung cancers. Nature *489*, 519-525.

Carter, S. L., Cibulskis, K., Helman, E., McKenna, A., Shen, H., Zack, T., Laird, P. W., Onofrio, R. C., Winckler, W., Weir, B. A.*, et al.* (2012). Absolute quantification of somatic DNA alterations in human cancer. Nat Biotechnol *30*, 413-421.

Chu, A., Robertson, G., Brooks, D., Mungall, A. J., Birol, I., Coope, R., Ma, Y., Jones, S., and Marra, M. A. (2016). Large-scale profiling of microRNAs for The Cancer Genome Atlas. Nucleic Acids Res *44*, e3.

Chu, J., Sadeghi, S., Raymond, A., Jackman, S. D., Nip, K. M., Mar, R., Mohamadi, H., Butterfield, Y. S., Robertson, A. G., and Birol, I. (2014). BioBloom tools: fast, accurate and memory-efficient host species sequence screening using bloom filters. Bioinformatics *30*, 3402-3404.

Dabney, A. R. (2006). ClaNC: point-and-click software for classifying microarrays to nearest centroids. Bioinformatics *22*, 122-123.

de Hoon, M. J., Imoto, S., Nolan, J., and Miyano, S. (2004). Open source clustering software. Bioinformatics *20*, 1453-1454.

Faith, J. J., Hayete, B., Thaden, J. T., Mogno, I., Wierzbowski, J., Cottarel, G., Kasif, S., Collins, J. J., and Gardner, T. S. (2007). Large-scale mapping and validation of Escherichia coli transcriptional regulation from a compendium of expression profiles. PLoS biology *5*, e8.

Forbes, S. A., Bindal, N., Bamford, S., Cole, C., Kok, C. Y., Beare, D., Jia, M., Shepherd, R., Leung, K., Menzies, A.*, et al.* (2011). COSMIC: mining complete cancer genomes in the Catalogue of Somatic Mutations in Cancer. Nucleic Acids Res *39*, D945-950.

Gaujoux, R., and Seoighe, C. (2010). A flexible R package for nonnegative matrix factorization. BMC Bioinformatics *11*, 367.

Ge, H., Liu, K., Juan, T., Fang, F., Newman, M., and Hoeck, W. (2011). FusionMap: detecting fusion genes from next-generation sequencing data at base-pair resolution. Bioinformatics *27*, 1922-1928.

Gonzalez-Angulo, A. M., Hennessy, B. T., Meric-Bernstam, F., Sahin, A., Liu, W., Ju, Z., Carey, M. S., Myhre, S., Speers, C., Deng, L., *et al.* (2011). Functional proteomics can define prognosis and predict pathologic complete response in patients with breast cancer. Clin Proteomics *8*, 11.

Hennessy, B. T., Lu, Y., Gonzalez-Angulo, A. M., Carey, M. S., Myhre, S., Ju, Z., Davies, M. A., Liu, W., Coombes, K., Meric-Bernstam, F., *et al.* (2010). A Technical Assessment of the Utility of Reverse Phase Protein Arrays for the Study of the Functional Proteome in Non-microdissected Human Breast Cancers. Clin Proteomics *6*, 129-151.

Hennessy, B. T., Lu, Y., Poradosu, E., Yu, Q., Yu, S., Hall, H., Carey, M. S., Ravoori, M., Gonzalez-Angulo, A. M., Birch, R., *et al.* (2007). Pharmacodynamic markers of perifosine efficacy. Clin Cancer Res *13*, 7421-7431.

Hsu, S. D., Tseng, Y. T., Shrestha, S., Lin, Y. L., Khaleel, A., Chou, C. H., Chu, C. F., Huang, H. Y., Lin, C. M., Ho, S. Y., *et al.* (2014). miRTarBase update 2014: an information resource for experimentally validated miRNA-target interactions. Nucleic Acids Res *42*, D78-85.

Hu, J., He, X., Baggerly, K. A., Coombes, K. R., Hennessy, B. T., and Mills, G. B. (2007). Non-parametric quantification of protein lysate arrays. Bioinformatics *23*, 1986-1994.

Kent, W. J. (2002). BLAT--the BLAST-like alignment tool. Genome Res *12*, 656-664.

Kim, D., Pertea, G., Trapnell, C., Pimentel, H., Kelley, R., and Salzberg, S. L. (2013). TopHat2: accurate alignment of transcriptomes in the presence of insertions, deletions and gene fusions. Genome Biol *14*, R36.

Kim, D., and Salzberg, S. L. (2011). TopHat-Fusion: an algorithm for discovery of novel fusion transcripts. Genome Biol *12*, R72.

Korn, J. M., Kuruvilla, F. G., McCarroll, S. A., Wysoker, A., Nemesh, J., Cawley, S., Hubbell, E., Veitch, J., Collins, P. J., Darvishi, K., *et al.* (2008). Integrated genotype calling and association analysis of SNPs, common copy number polymorphisms and rare CNVs. Nature genetics *40*, 1253-1260.

Kostic, A. D., Ojesina, A. I., Pedamallu, C. S., Jung, J., Verhaak, R. G., Getz, G., and Meyerson, M. (2011). PathSeq: software to identify or discover microbes by deep sequencing of human tissue. Nat Biotechnol *29*, 393-396.

Kuhn, R. M., Haussler, D., and Kent, W. J. (2013). The UCSC genome browser and associated tools. Brief Bioinform *14*, 144-161.

Lawrence, M. S., Stojanov, P., Polak, P., Kryukov, G. V., Cibulskis, K., Sivachenko, A., Carter, S. L., Stewart, C., Mermel, C. H., Roberts, S. A.*, et al.* (2013). Mutational heterogeneity in cancer and the search for new cancer-associated genes. Nature *499*, 214-218.

Leek, J. T., Johnson, W. E., Parker, H. S., Jaffe, A. E., and Storey, J. D. (2012). The sva package for removing batch effects and other unwanted variation in high-throughput experiments. Bioinformatics *28*, 882-883.

Li, B., and Dewey, C. N. (2011). RSEM: accurate transcript quantification from RNA-Seq data with or without a reference genome. BMC Bioinformatics *12*, 323.

Li, H., and Durbin, R. (2009). Fast and accurate short read alignment with Burrows-Wheeler transform. Bioinformatics *25*, 1754-1760.

Li, J., and Tibshirani, R. (2013). Finding consistent patterns: a nonparametric approach for identifying differential expression in RNA-Seq data. Stat Methods Med Res *22*, 519-536.

Liang, J., Shao, S. H., Xu, Z. X., Hennessy, B., Ding, Z., Larrea, M., Kondo, S., Dumont, D. J., Gutterman, J. U., Walker, C. L.*, et al.* (2007). The energy sensing LKB1-AMPK pathway regulates p27(kip1) phosphorylation mediating the decision to enter autophagy or apoptosis. Nat Cell Biol *9*, 218-224.

McCarroll, S. A., Kuruvilla, F. G., Korn, J. M., Cawley, S., Nemesh, J., Wysoker, A., Shapero, M. H., de Bakker, P. I., Maller, J. B., Kirby, A.*, et al.* (2008). Integrated detection and population-genetic analysis of SNPs and copy number variation. Nature genetics *40*, 1166-1174.

McPherson, A., Hormozdiari, F., Zayed, A., Giuliany, R., Ha, G., Sun, M. G., Griffith, M., Heravi Moussavi, A., Senz, J., Melnyk, N.*, et al.* (2011). deFuse: an algorithm for gene fusion discovery in tumor RNA-Seq data. PLoS Comput Biol *7*, e1001138.

Mermel, C. H., Schumacher, S. E., Hill, B., Meyerson, M. L., Beroukhim, R., and Getz, G. (2011). GISTIC2.0 facilitates sensitive and confident localization of the targets of focal somatic copy-number alteration in human cancers. Genome Biol *12*, R41.

Mullokandov, G., Baccarini, A., Ruzo, A., Jayaprakash, A. D., Tung, N., Israelow, B., Evans, M. J., Sachidanandam, R., and Brown, B. D. (2012). High-throughput assessment of microRNA activity and function using microRNA sensor and decoy libraries. Nat Methods *9*, 840-846.

Olshen, A. B., Venkatraman, E. S., Lucito, R., and Wigler, M. (2004). Circular binary segmentation for the analysis of array-based DNA copy number data. Biostatistics *5*, 557-572.

Pascual-Montano, A., Carazo, J. M., Kochi, K., Lehmann, D., and Pascual-Marqui, R. D. (2006). Nonsmooth nonnegative matrix factorization (nsNMF). IEEE Trans Pattern Anal Mach Intell *28*, 403-415.

Robertson, G., Schein, J., Chiu, R., Corbett, R., Field, M., Jackman, S. D., Mungall, K., Lee, S., Okada, H. M., Qian, J. Q.*, et al.* (2010). De novo assembly and analysis of RNA-seq data. Nat Methods *7*, 909-912.

Robinson, J. T., Thorvaldsdottir, H., Winckler, W., Guttman, M., Lander, E. S., Getz, G., and Mesirov, J. P. (2011). Integrative genomics viewer. Nat Biotechnol *29*, 24-26.

Saeed, A. I., Sharov, V., White, J., Li, J., Liang, W., Bhagabati, N., Braisted, J., Klapa, M., Currier, T., Thiagarajan, M.*, et al.* (2003). TM4: a free, open-source system for microarray data management and analysis. Biotechniques *34*, 374-378.

Saldanha, A. J. (2004). Java Treeview--extensible visualization of microarray data. Bioinformatics *20*, 3246-3248.

Shen, Y., Wan, Z., Coarfa, C., Drabek, R., Chen, L., Ostrowski, E. A., Liu, Y., Weinstock, G. M., Wheeler, D. A., Gibbs, R. A., and Yu, F. (2010). A SNP discovery method to assess variant allele probability from next-generation resequencing data. Genome Res *20*, 273-280.

Sherry, S. T., Ward, M. H., Kholodov, M., Baker, J., Phan, L., Smigielski, E. M., and Sirotkin, K. (2001). dbSNP: the NCBI database of genetic variation. Nucleic Acids Res *29*, 308-311.

Tay, Y., Rinn, J., and Pandolfi, P. P. (2014). The multilayered complexity of ceRNA crosstalk and competition. Nature *505*, 344-352.

Thorvaldsdottir, H., Robinson, J. T., and Mesirov, J. P. (2013). Integrative Genomics Viewer (IGV): high-performance genomics data visualization and exploration. Brief Bioinform *14*, 178-192.

Tibes, R., Qiu, Y., Lu, Y., Hennessy, B., Andreeff, M., Mills, G. B., and Kornblau, S. M. (2006). Reverse phase protein array: validation of a novel proteomic technology and utility for analysis of primary leukemia specimens and hematopoietic stem cells. Mol Cancer Ther *5*, 2512-2521.

Torres-Garcia, W., Zheng, S., Sivachenko, A., Vegesna, R., Wang, Q., Yao, R., Berger, M. F., Weinstein, J. N., Getz, G., and Verhaak, R. G. (2014). PRADA: pipeline for RNA sequencing data analysis. Bioinformatics *30*, 2224-2226.

Trapnell, C., Williams, B. A., Pertea, G., Mortazavi, A., Kwan, G., van Baren, M. J., Salzberg, S. L., Wold, B. J., and Pachter, L. (2010). Transcript assembly and quantification by RNA-Seq reveals unannotated transcripts and isoform switching during cell differentiation. Nat Biotechnol *28*, 511-515.

Wang, K., Li, M., and Hakonarson, H. (2010a). ANNOVAR: functional annotation of genetic variants from high-throughput sequencing data. Nucleic Acids Res *38*, e164.

Wang, K., Singh, D., Zeng, Z., Coleman, S. J., Huang, Y., Savich, G. L., He, X., Mieczkowski, P., Grimm, S. A., Perou, C. M.*, et al.* (2010b). MapSplice: accurate mapping of RNA-seq reads for splice junction discovery. Nucleic Acids Res *38*, e178.

Ward, J. J. H. (1963). Hierarchical grouping to optimize an objective function. Journal of American statistical association *58*, 236-244.

Wilkerson, M. D., and Hayes, D. N. (2010). ConsensusClusterPlus: a class discovery tool with confidence assessments and item tracking. Bioinformatics *26*, 1572-1573.

Wilks, C., Cline, M. S., Weiler, E., Diehkans, M., Craft, B., Martin, C., Murphy, D., Pierce, H., Black, J., Nelson, D.*, et al.* (2014). The Cancer Genomics Hub (CGHub): overcoming cancer through the power of torrential data. Database (Oxford) *2014*.

Wu, C. C., Kannan, K., Lin, S., Yen, L., and Milosavljevic, A. (2013). Identification of cancer fusion drivers using network fusion centrality. Bioinformatics *29*, 1174-1181.

Ye, K., Schulz, M. H., Long, Q., Apweiler, R., and Ning, Z. (2009). Pindel: a pattern growth approach to detect break points of large deletions and medium sized insertions from paired-end short reads. Bioinformatics *25*, 2865-2871.

Zack, T. I., Schumacher, S. E., Carter, S. L., Cherniack, A. D., Saksena, G., Tabak, B., Lawrence, M. S., Zhsng, C. Z., Wala, J., Mermel, C. H.*, et al.* (2013). Pan-cancer patterns of somatic copy number alteration. Nature genetics *45*, 1134-1140.

Supplemental Figure 1

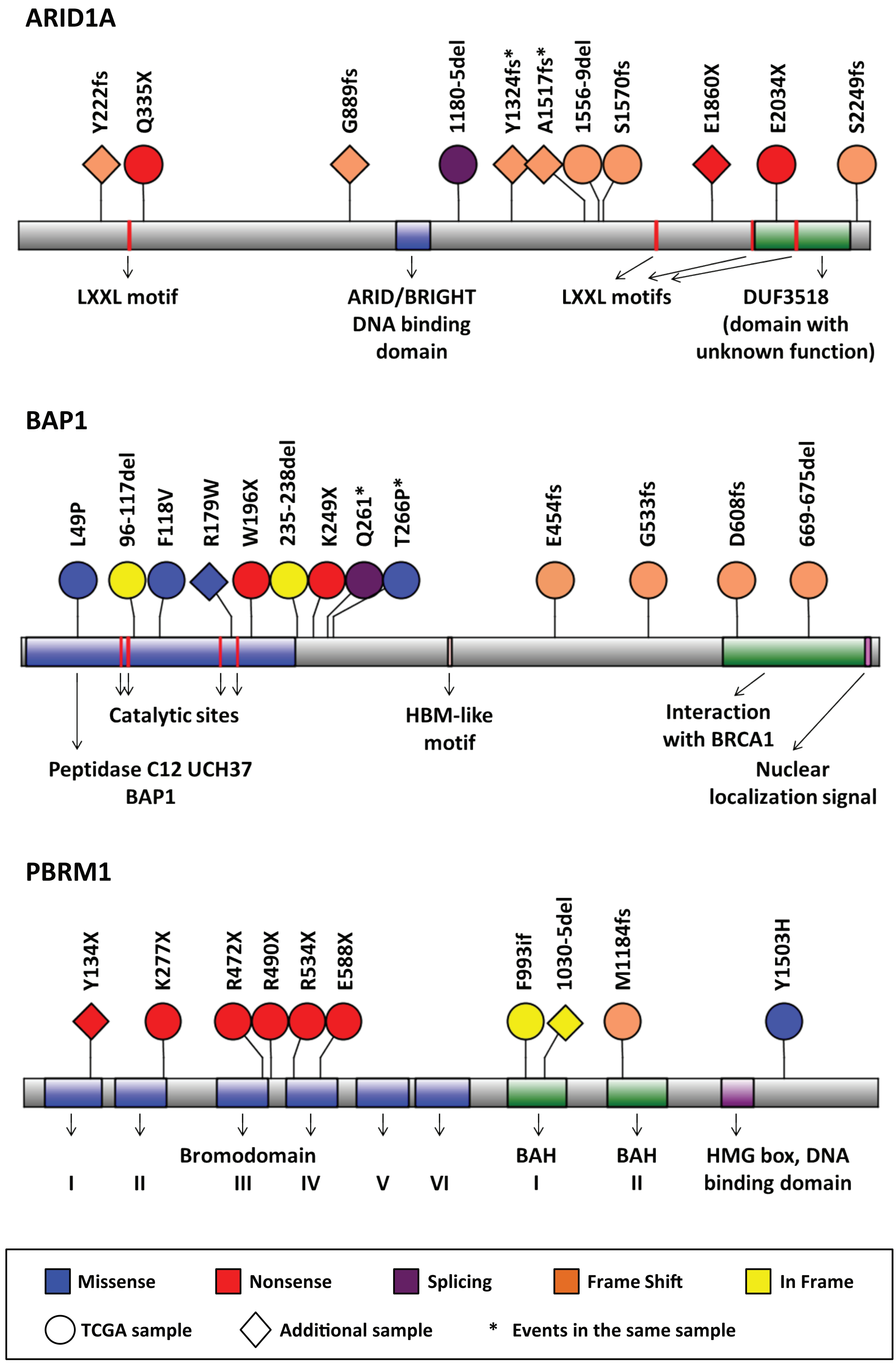

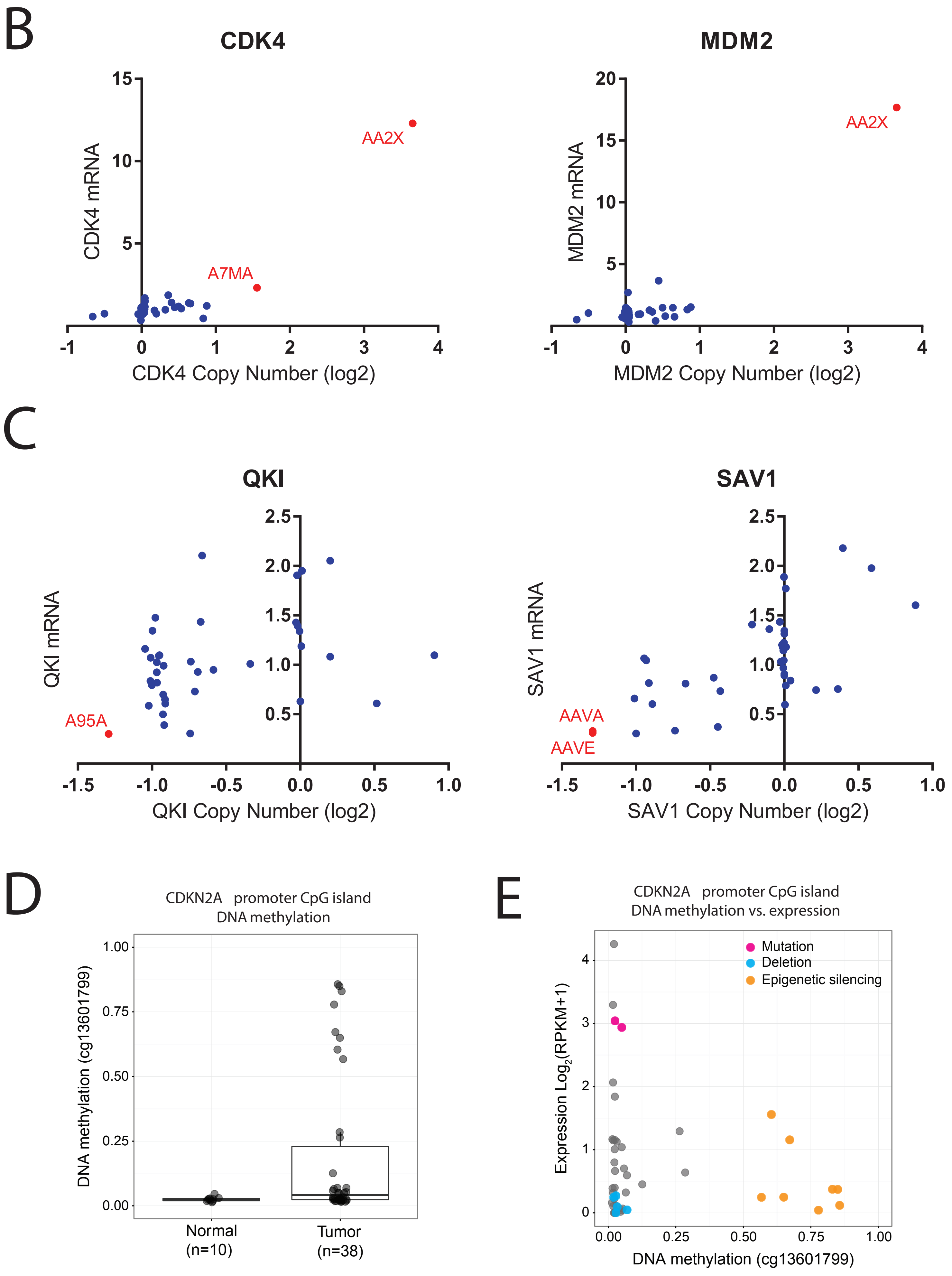

B
CDK4
CDK4 mRNA
CDK4 Copy Number (log2)
AA2X
A7MA
MDM2
MDM2 mRNA
MDM2 Copy Number (log2)
AA2X
C
QKI
QKI mRNA
QKI Copy Number (log2)
A95A
SAV1
SAV1 mRNA
SAV1 Copy Number (log2)
AAVA
AAVE
D
CDKN2A promoter CpG island
DNA methylation
DNA methylation (cg13601799)
Normal
(n=10)
Tumor
(n=38)
E
CDKN2A promoter CpG island
DNA methylation vs. expression
Mutation
Deletion
Epigenetic silencing
Expression Log2(RPKM+1)
DNA methylation (cg13601799)

F

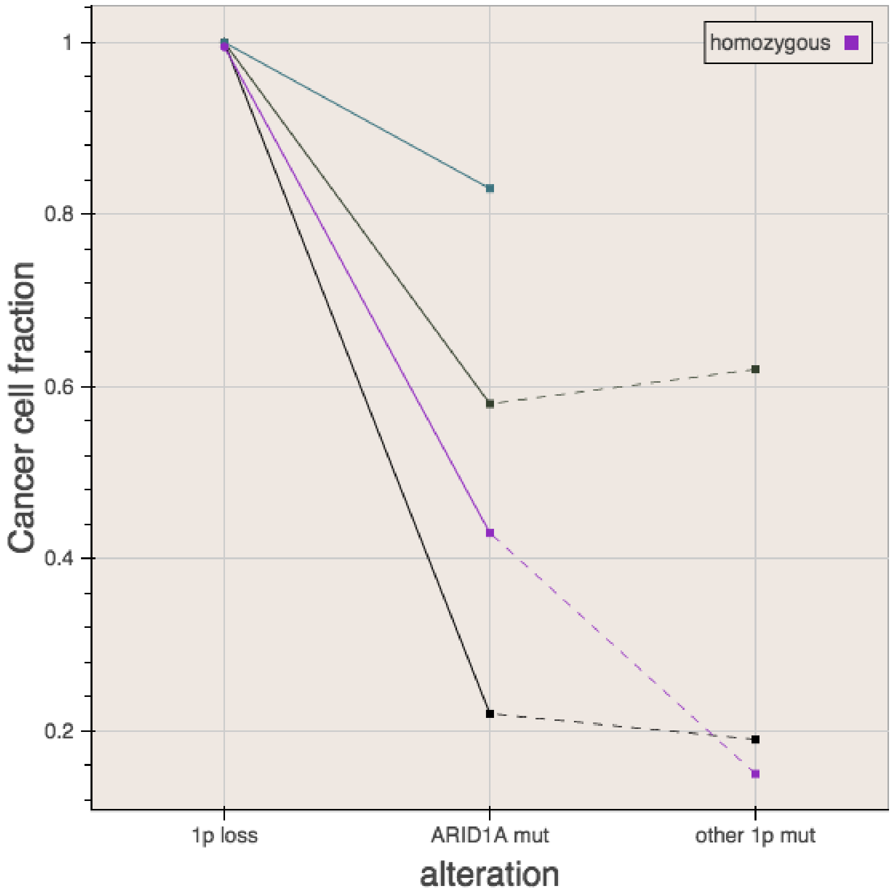

homozygous
1
0.8
0.6
0.4
0.2
Cancer cell fraction
1p loss
ARID1A mut
other 1p mut
alteration

G

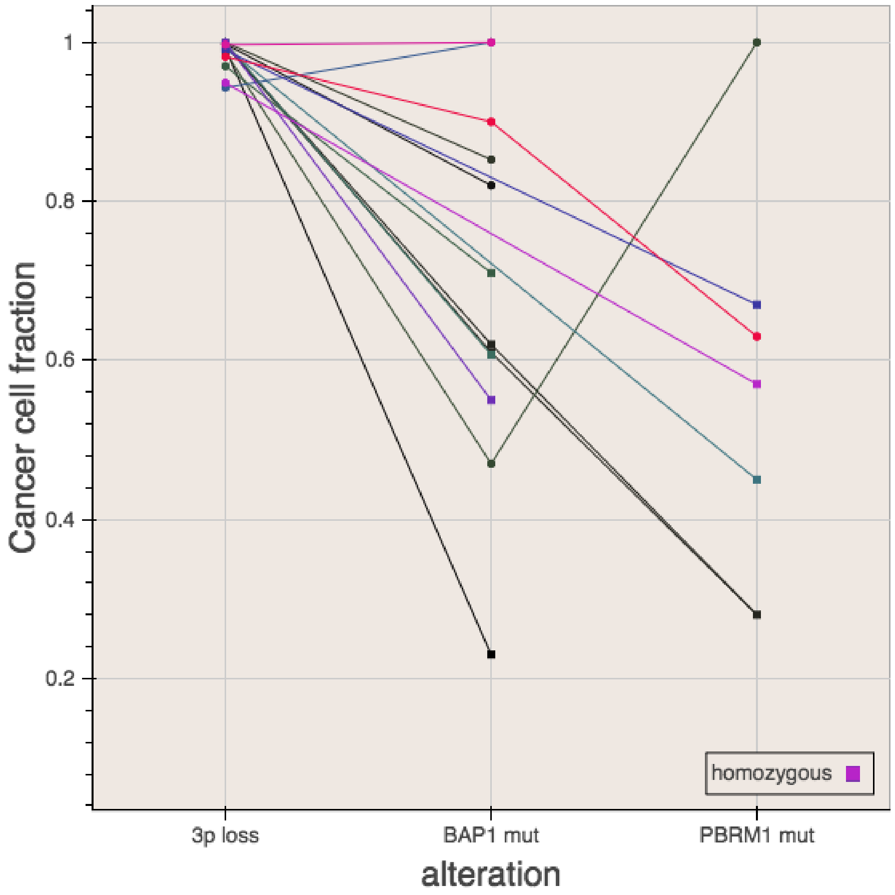

1
0.8
0.6
0.4
0.2
Cancer cell fraction
homozygous
3p loss
BAP1 mut
PBRM1 mut
alteration

Supplemental Figure 1. Somatic mutations and copy number alterations, related to Figure 1. A) Lollipop depictions of mutations identified in the tumor-suppressor genes *ARID1A, BAP1,* and *PBRM1*. B and C) Correlations between RNA and somatic copy number alterations or DNA methylation. B) mRNA correlations with copy number for *CDK4* and *MDM2* affected by focal amplification. C) mRNA correlations with copy number for *QKI* and *SAV1* affected by focal deletion. D) Hypermethylation at the *CDKN2A* promoter CpG island in tumors vs. normal tissues. E) mRNA correlations for *CDKN2A* exon 1 with *CDKN2A* promoter CpG island methylation. F and G) Cancer cell fractions for broad or arm-level loss of chromosomes vs. mutations of tumor suppressor genes on those arms. F) Chromosome 1p loss vs. *ARID1A* mutations. G) Chromsome 3p loss vs. *BAP1* and *PBRM1* mutations.

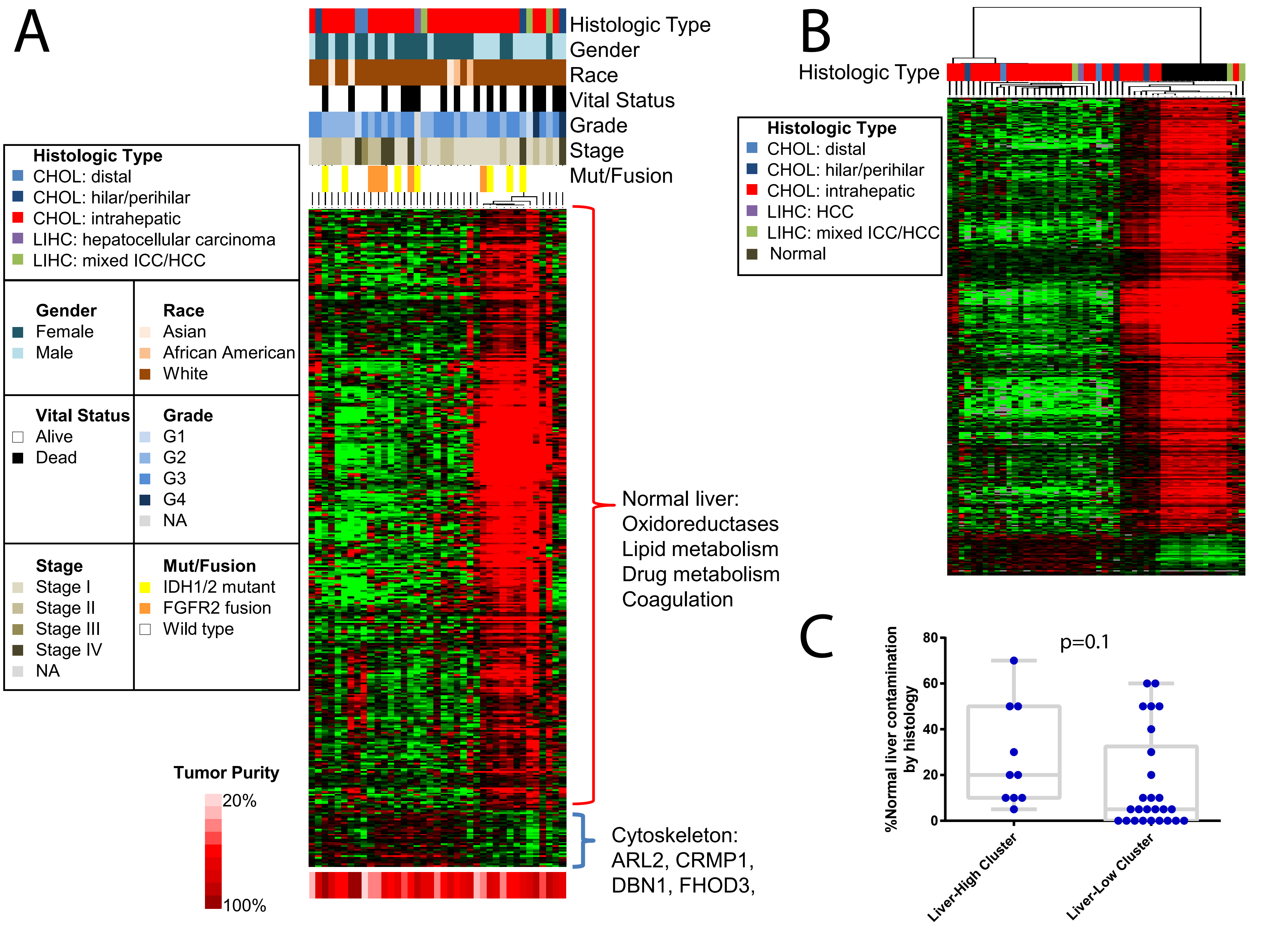

A
Histologic Type
Gender
Race
Vital Status
Grade
Stage
Mut/Fusion
Histologic Type
CHOL: distal
CHOL: hilar/perihilar
CHOL: intrahepatic
LIHC: hepatocellular carcinoma
LIHC: mixed ICC/HCC
Gender
Female
Male
Race
Asian
African American
White
Vital Status
Alive
Dead
Grade
G1
G2
G3
G4
NA
Stage
Stage I
Stage II
Stage III
Stage IV
NA
Mut/Fusion
IDH1/2 mutant
FGFR2 fusion
Wild type
Tumor Purity
20%
100%
Normal liver:
Oxidoreductases
Lipid metabolism
Drug metabolism
Coagulation
Cytoskeleton:
ARL2, CRMP1,
DBN1, FHOD3,
B
Histologic Type
Histologic Type
CHOL: distal
CHOL: hilar/perihilar
CHOL: intrahepatic
LIHC: HCC
LIHC: mixed ICC/HCC
Normal
C
p=0.1
%Normal liver contamination by histology
0
20
40
60
80
Liver-High Cluster
Liver-Low Cluster

Supplemental Figure 2. Initial mRNA clustering of the 38 CCA samples, using the top 400 most-variable genes, related to Figure 2. A) Clustering of only the tumors, which identified liver genes as the dominant determining signature. B) Clustering of tumors plus 11 normal liver samples. C) Percent liver contamination of all tumor samples as assessed from nearby hematoxylin and eosin–stained histology slides.

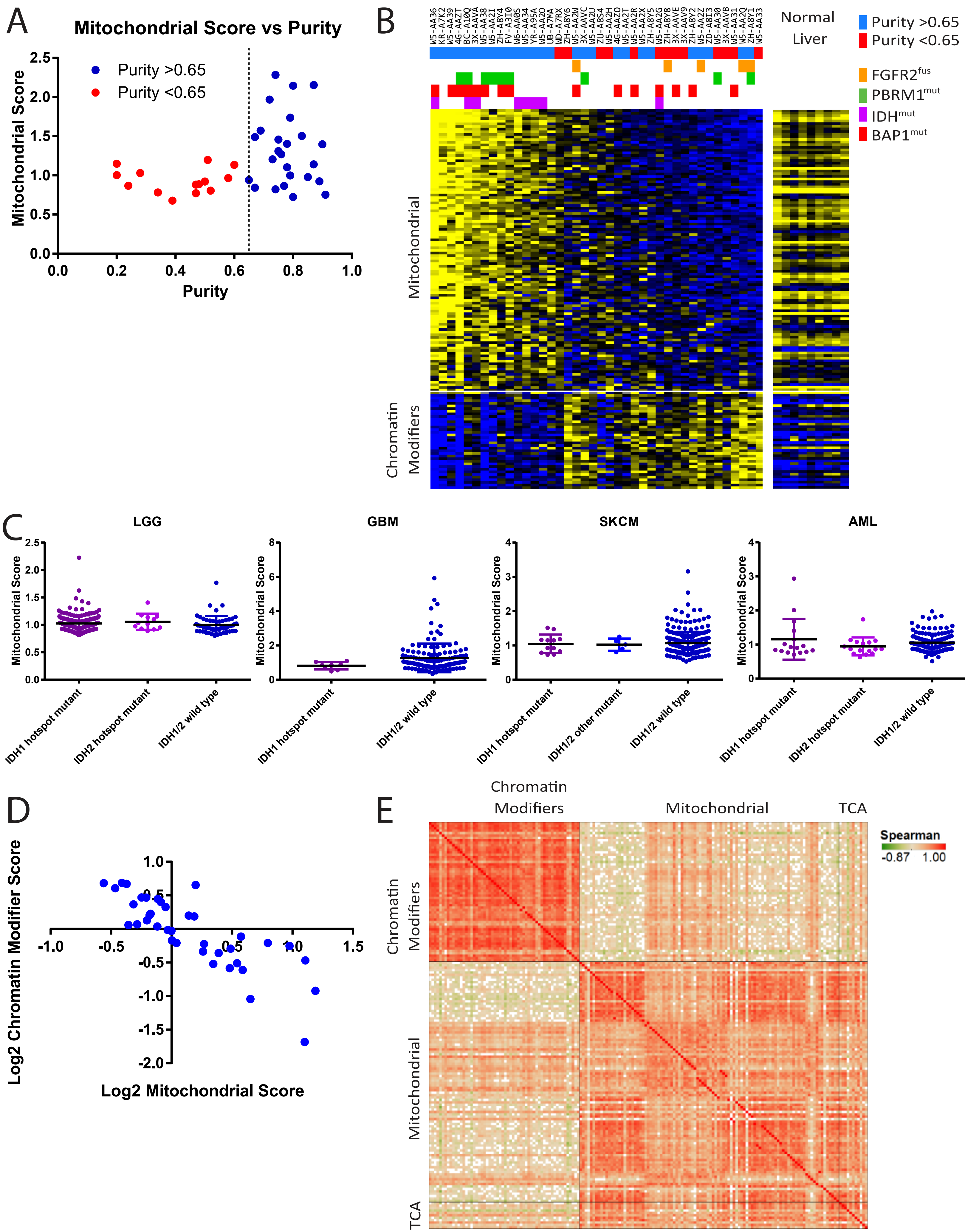
A
Mitochondrial Score vs Purity
Purity >0.65
Purity <0.65
Mitochondrial Score
Purity
B
Normal Liver
Purity >0.65
Purity <0.65
FGFR2fus
PBRM1mut
IDHmut
BAP1mut
Mitochondrial
Chromatin Modifiers
C
LGG
GBM
SKCM
AML
Mitochondrial Score
IDH1 hotspot mutant
IDH2 hotspot mutant
IDH1/2 wild type
IDH1/2 other mutant
D
Log2 Chromatin Modifier Score
Log2 Mitochondrial Score
E
Chromatin Modifiers
Mitochondrial
TCA
Spearman
-0.87
1.00

Supplemental Figure 3. Characteristics of mitochondrial and chromatin modifier signatures, related to Figure 3. A) Low tumor purity correlates with a low mitochondrial score in the CHOL cohort. B) *IDH* mutations associate with a high mitochondrial score and *FGFR2* fusions with a low OXPHOS score regardless of tumor purity. C) Mitochondrial scores do not correlate with *IDH* mutation status in glioblastoma (GBM), low grade glioma (LGG), melanoma (SKCM), or acute myeloid leukemia (AML) in TCGA datasets. D) OXPHOS and chromatin modifier scores anticorrelated along a gradient and E) across normal tissues in the GTEx database. Red indicates correlation, green indicates anticorrelation.

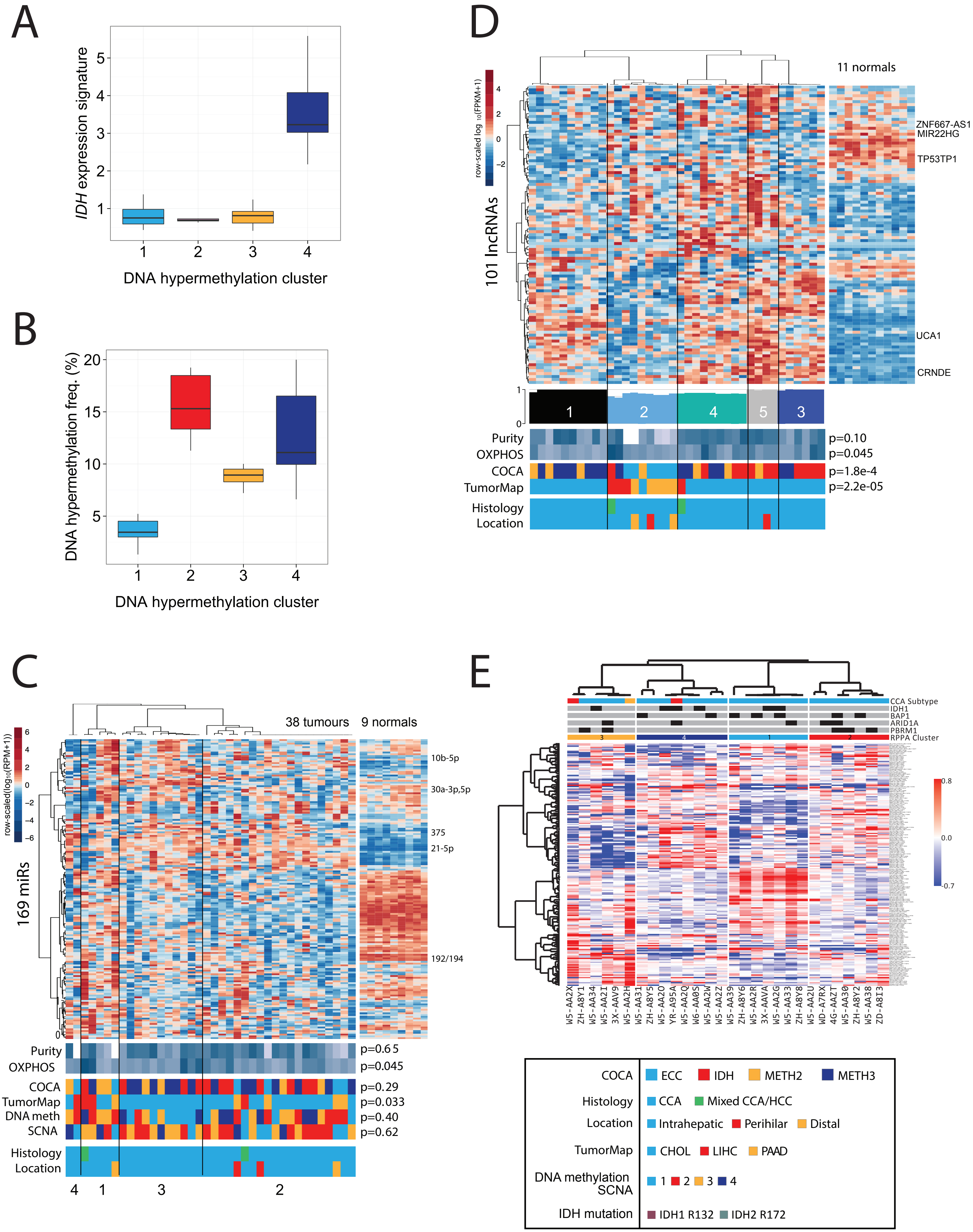

A
IDH expression signature
DNA hypermethylation cluster
B
DNA hypermethylation freq. (%)
DNA hypermethylation cluster
C
row-scaled(log10(RPM+1))
169 miRs
38 tumours
9 normals
10b-5p
30a-3p,5p
375
21-5p
192/194
Purity
OXPHOS
COCA
TumorMap
DNA meth
SCNA
Histology
Location
p=0.65
p=0.045
p=0.29
p=0.033
p=0.40
p=0.62
4
1
3
2
D
row-scaled log 10(FPKM+1)
101 lncRNAs
11 normals
ZNF667-AS1
MIR22HG
TP53TP1
UCA1
CRNDE
Purity
OXPHOS
COCA
TumorMap
Histology
Location
p=0.10
p=0.045
p=1.8e-4
p=2.2e-05
E
CCA Subtype
IDH1
BAP1
ARID1A
PBRM1
RPPA Cluster
COCA
ECC
IDH
METH2
METH3
Histology
CCA
Mixed CCA/HCC
Location
Intrahepatic
Perihilar
Distal
TumorMap
CHOL
LIHC
PAAD
DNA methylation
SCNA
IDH mutation
IDH1 R132
IDH2 R172

Supplemental Figure 4. DNA methylation, MicroRNA, lncRNA, and reverse-phase protein array analyses, related to Figure 4. A) *IDH* signature scores (Supplemental Table 2) for each sample from each cluster. B) DNA hypermethylation frequencies for each sample from each cluster. C-D) Multiplatform unsupervised cluster analysis of cholangiocarcinoma for C) miRNA expression, D) lncRNA expression, and E) RPPA. The miRNA and lncRNA clusters were identified after removal of normal liver–specific transcripts. No normal liver–specific proteins were identified for RPPA.

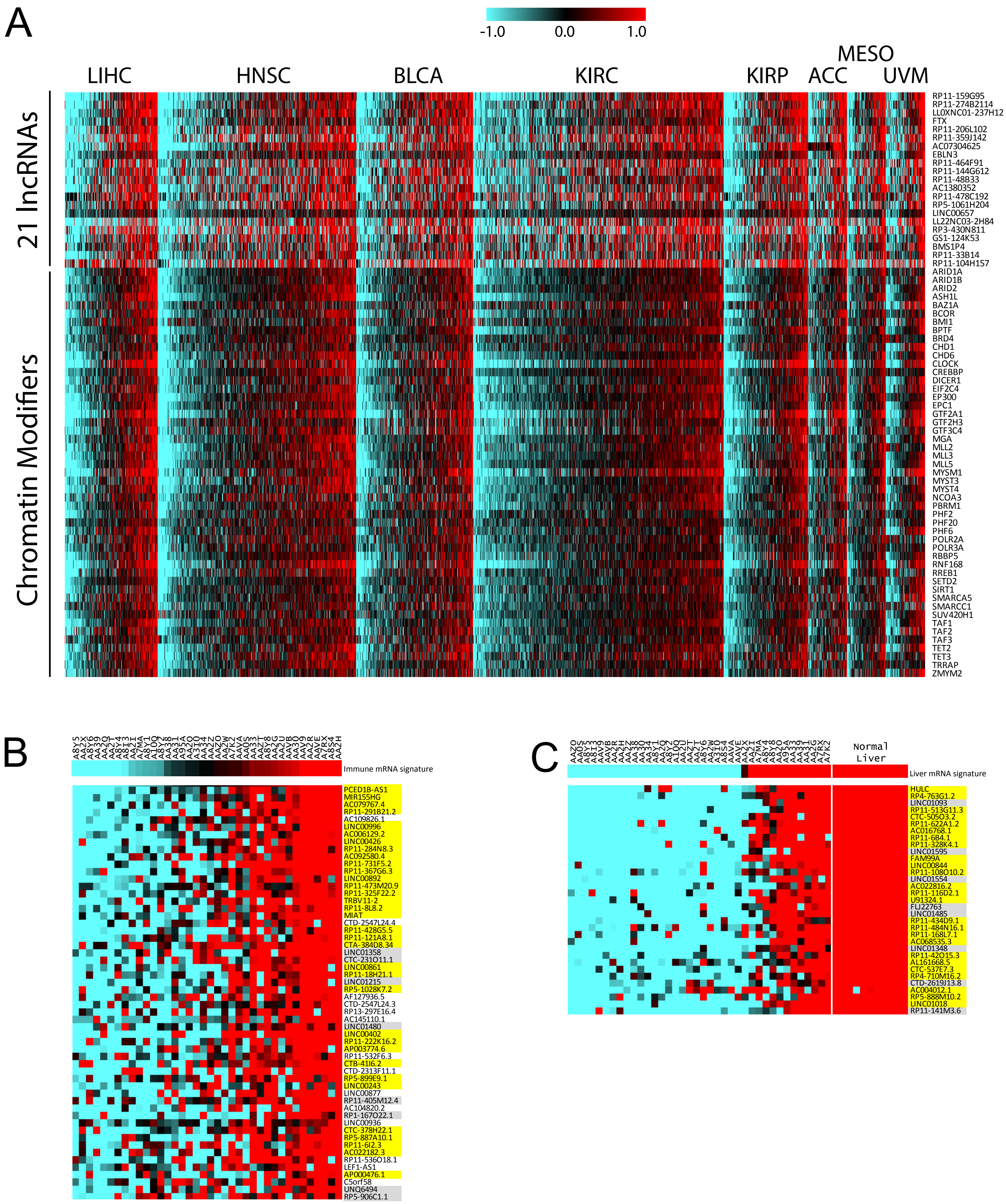

A
-1.0
0.0
1.0
LIHC
HNSC
BLCA
KIRC
KIRP
ACC
MESO
UVM
21 lncRNAs
Chromatin Modifiers
RP11-159G95
RP11-274B2114
LLOXNC01-237H12
FTX
RP11-206L102
RP11-359J142
AC07304625
EBLN3
RP11-464F91
RP11-144G612
RP11-48B33
AC1380352
RP11-478C192
RP5-1061H204
LINC00657
LL22NC03-2H84
RP3-430N811
GS1-124K53
BMS1P4
RP11-33B14
RP11-104H157
ARID1A
ARID1B
ARID2
ASH1L
BAZ1A
BCOR
BMI1
BPTF
BRD4
CHD1
CHD6
CLOCK
CREBBP
DICER1
EIF2C4
EP300
EPC1
GTF2A1
GTF2H3
GTF3C4
MGA
MLL2
MLL3
MLL5
MYSM1
MYST3
MYST4
NCOA3
PBRM1
PHF2
PHF20
PHF6
POLR2A
POLR3A
RBBP5
RNF168
RREB1
SETD2
SIRT1
SMARCA5
SMARCC1
SUV420H1
TAF1
TAF2
TAF3
TET2
TET3
TRRAP
ZMYM2
B
Immune mRNA signature
PCED1B-AS1
MIR155HG
AC079767.4
RP11-291B21.2
AC109826.1
LINC00996
AC006129.2
LINC00426
RP11-284N8.3
AC092580.4
RP11-731F5.2
RP11-367G6.3
LINC00892
RP11-473M20.9
RP11-325F22.2
TRBV11-2
RP11-8L8.2
MIAT
CTD-2547L24.4
RP11-428G5.5
RP11-121A8.1
CTA-384D8.34
LINC01358
CTC-231O11.1
LINC00861
RP11-18H21.1
LINC01215
RP5-1028K7.2
AF127936.5
CTD-2547L24.3
RP13-297E16.4
AC145110.1
LINC01480
LINC00402
RP11-222K16.2
AP003774.6
RP11-532F6.3
CTB-41I6.2
CTD-2313F11.1
RP5-899E9.1
LINC00243
LINC00877
RP11-405M12.4
AC104820.2
RP1-167O22.1
LINC00936
CTC-378H22.1
RP5-887A10.1
RP11-6I2.3
AC022182.3
RP11-536O18.1
LEF1-AS1
AP000476.1
C5orf58
UNQ6494
RP5-906C1.1
C
Normal Liver
Liver mRNA signature
HULC
RP4-763G1.2
LINC01093
RP11-513G11.3
CTC-505O3.2
RP11-622A1.2
AC016768.1
RP11-6B4.1
RP11-328K4.1
LINC01595
FAM99A
LINC00844
RP11-108O10.2
LINC01554
AC022816.2
RP11-116D2.1
U91324.1
FLJ22763
LINC01485
RP11-434D9.1
RP11-484N16.1
RP11-168L7.1
AC068535.3
LINC01348
RP11-42O15.3
AL161668.5
CTC-537E7.3
RP4-710M16.2
CTD-2619J13.8
AC004012.1
RP5-888M10.2
LINC01018
RP11-141M3.6

D

miRs that are differentially expressed in IDH COC2 vs COC3/4

n	miR	SAM score	SAM q-val %	mean RPM IDH	mean RPM METH2,3	FC: IDH vs METH2,3	-1/FC	Wilcoxon P (uncorr)	Wilcoxon P(BH)
1	miR-96-5p	1.70	0.0	37.4	22.8	1.64	-0.608	0.000346	0.013
2	miR-194-5p	1.44	0.0	8,011.9	3,927.2	2.04	-0.49	0.00314	0.04
3	let-7f-5p	1.32	0.0	9,531.9	5,516.5	1.73	-0.579	0.0073	0.056
4	miR-34a-5p	1.32	0.0	404.0	225.7	1.79	-0.559	0.0073	0.056
5	miR-182-5p	1.18	0.0	20,798.7	11,998.7	1.73	-0.577	0.0175	0.083
1	miR-135b-5p	-1.58	0.0	7.7	46.6	0.165	-6.076	0.00103	0.0196
2	miR-375	-1.26	5.3	4,558.4	19,354.0	0.236	-4.246	0.0108	0.0684
3	miR-187-3p	-1.18	5.3	13.2	26.6	0.496	-2.017	0.0175	0.0831

F

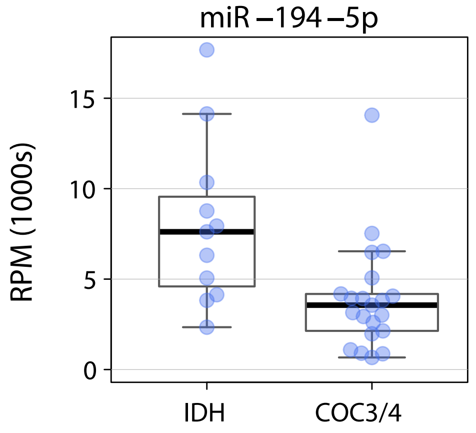

E

miRs that are significantly correlated with mitochondrial score

n	miR	rho	P	FDR	mn(RPM) T	mn(RPM) N	FC (T vs N)	-1/FC
1	miR-194-5p	0.51	0.00096	0.083	**4,914.9**	18,552.6	0.27	-3.78
2	miR-133a-3p	-0.53	0.00061	0.071	6.8	6.3	1.08	-0.93
3	miR-145-3p	-0.53	0.00060	0.071	44.7	49.3	0.91	-1.10
4	miR-145-5p	-0.55	0.00035	0.071	**1,682.5**	2,047.0	0.82	-1.22

G

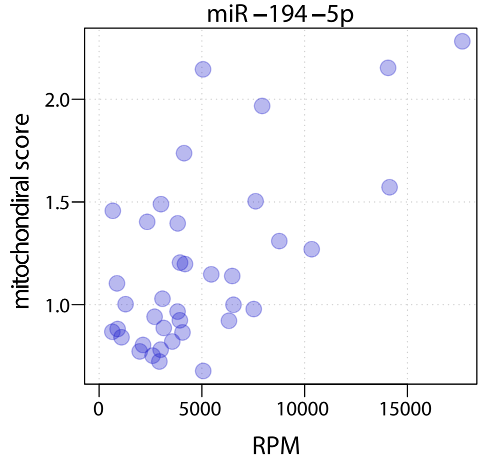

H

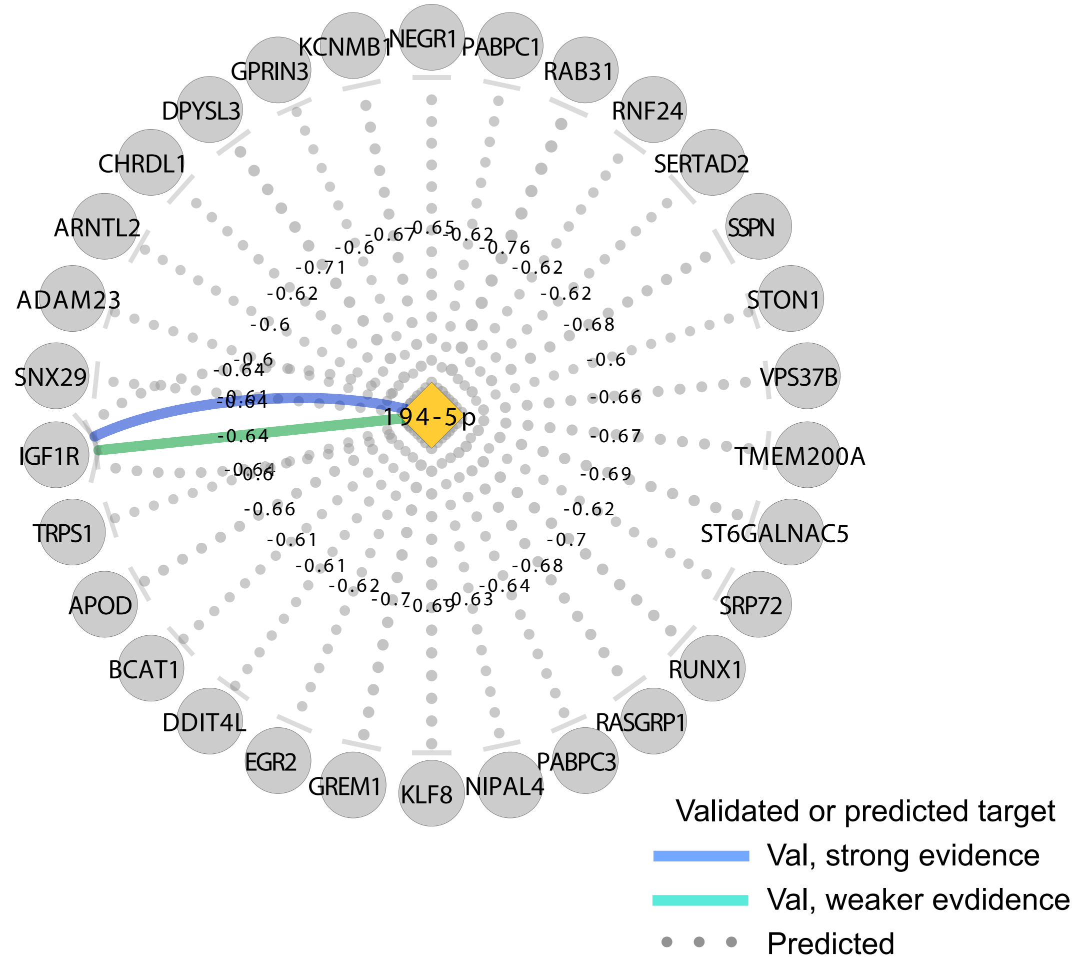

Supplemental Figure 5. Specific lncRNAs and miR-194-5p correlate with the chromatin modifier signature, related to Figure 5. A) 21 lncRNAs that correlate with the chromatin modifier mRNA signature across cancers. Two lncRNAs, EBLN3 and LINC00657 appear darker due to a smaller dynamic range. B) and C) lncRNAs that correlate with the immune and liver signatures. lncRNAs in yellow overlap with GTEx-derived immune/liver lists, while those in gray were not annotated in the GTEx database. Those in white do not overlap with the GTEx lists. D) miRs enriched in the IDH mutant COCA2. E) miRs most highly correlated with mitochondrial scores. F) miR-194 expression in COCA2 vs COCA3+4. G) miR-194 expression vs. mitochondrial scores. H) Predicted and functionally validated targets of miR-194. Text on subnetwork edges gives the Spearman correlation coefficients between miRNA and RSEM gene-level normalized abundance.

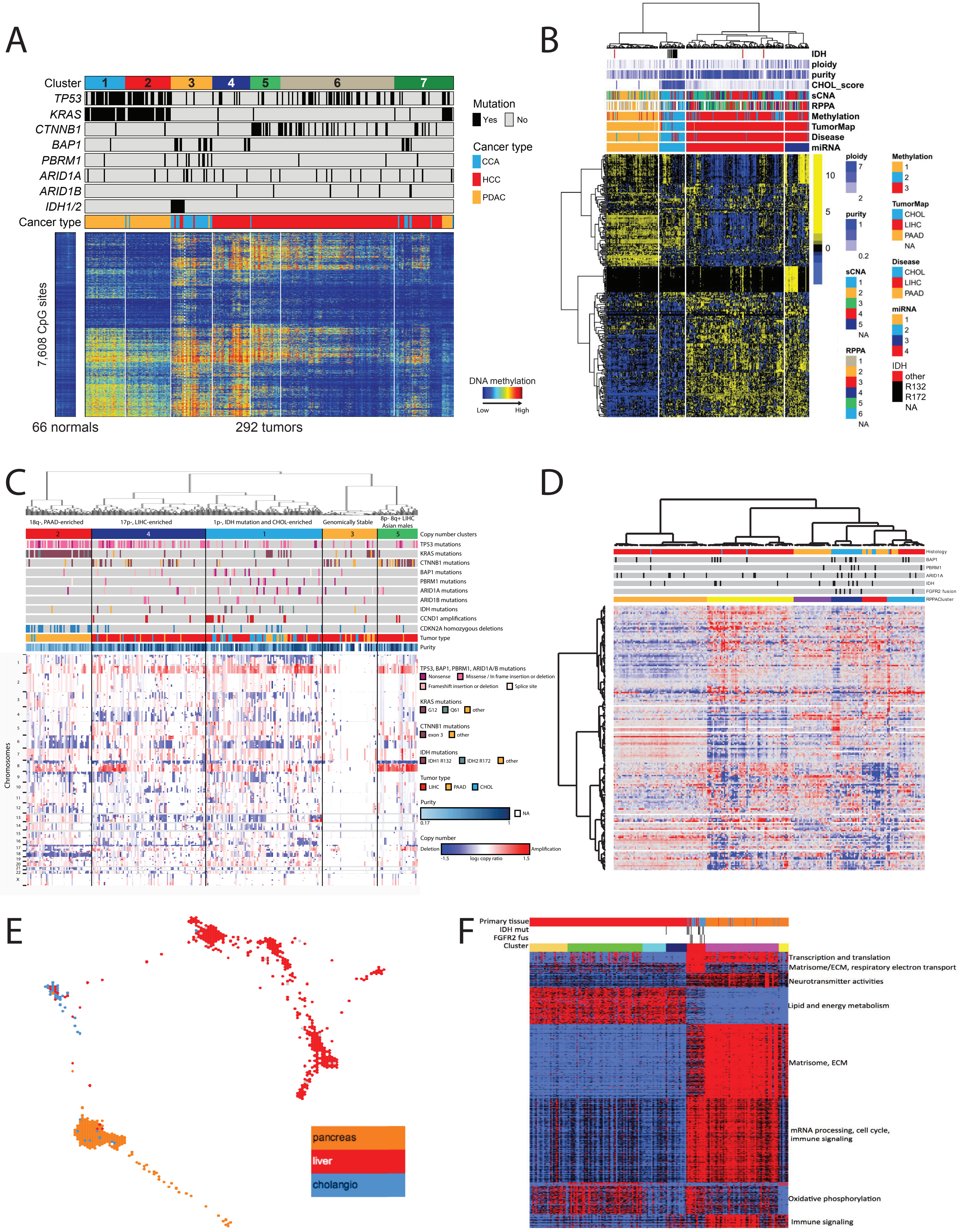

A
Cluster
1
2
3
4
5
6
7
TP53
KRAS
CTNNB1
BAP1
PBRM1
ARID1A
ARID1B
IDH1/2
Cancer type
7,608 CpG sites
66 normals
292 tumors
Mutation
Yes
No
Cancer type
CCA
HCC
PDAC
DNA methylation
Low
High
B
IDH
ploidy
purity
CHOL_score
sCNA
RPPA
Methylation
TumorMap
Disease
miRNA
10
5
0
ploidy
7
2
purity
1
0.2
sCNA
1
2
3
4
5
NA
RPPA
1
2
3
4
5
6
NA
Methylation
1
2
3
TumorMap
CHOL
LIHC
PAAD
NA
Disease
CHOL
LIHC
PAAD
miRNA
1
2
3
4
IDH
other
R132
R172
NA
C
18q-, PAAD-enriched
17p-, LIHC-enriched
1p-, IDH mutation and CHOL-enriched
Genomically Stable
8p- 8q+ LIHC Asian males
2
4
1
3
5
Copy number clusters
TP53 mutations
KRAS mutations
CTNNB1 mutations
BAP1 mutations
PBRM1 mutations
ARID1A mutations
ARID1B mutations
IDH mutations
CCND1 amplifications
CDKN2A homozygous deletions
Tumor type
Purity
Chromosomes
TP53, BAP1, PBRM1, ARID1A/B mutations
Nonsense
Missense / In frame insertion or deletion
Frameshift insertion or deletion
Splice site
KRAS mutations
G12
Q61
other
CTNNB1 mutations
exon 3
other
IDH mutations
IDH1 R132
IDH2 R172
other
Tumor type
LIHC
PAAD
CHOL
Purity
0.17
1
NA
Copy number
Deletion
Amplification
-1.5
log2 copy ratio
1.5
D
Histology
BAP1
PBRM1
ARID1A
IDH
FGFR2 fusion
RPPACluster
E
pancreas
liver
cholangio
F
Primary tissue
IDH mut
FGFR2 fus
Cluster
Transcription and translation
Matrisome/ECM, respiratory electron transport
Neurotransmitter activities
Lipid and energy metabolism
Matrisome, ECM
mRNA processing, cell cycle, immune signaling
Oxidative phosphorylation
Immune signaling

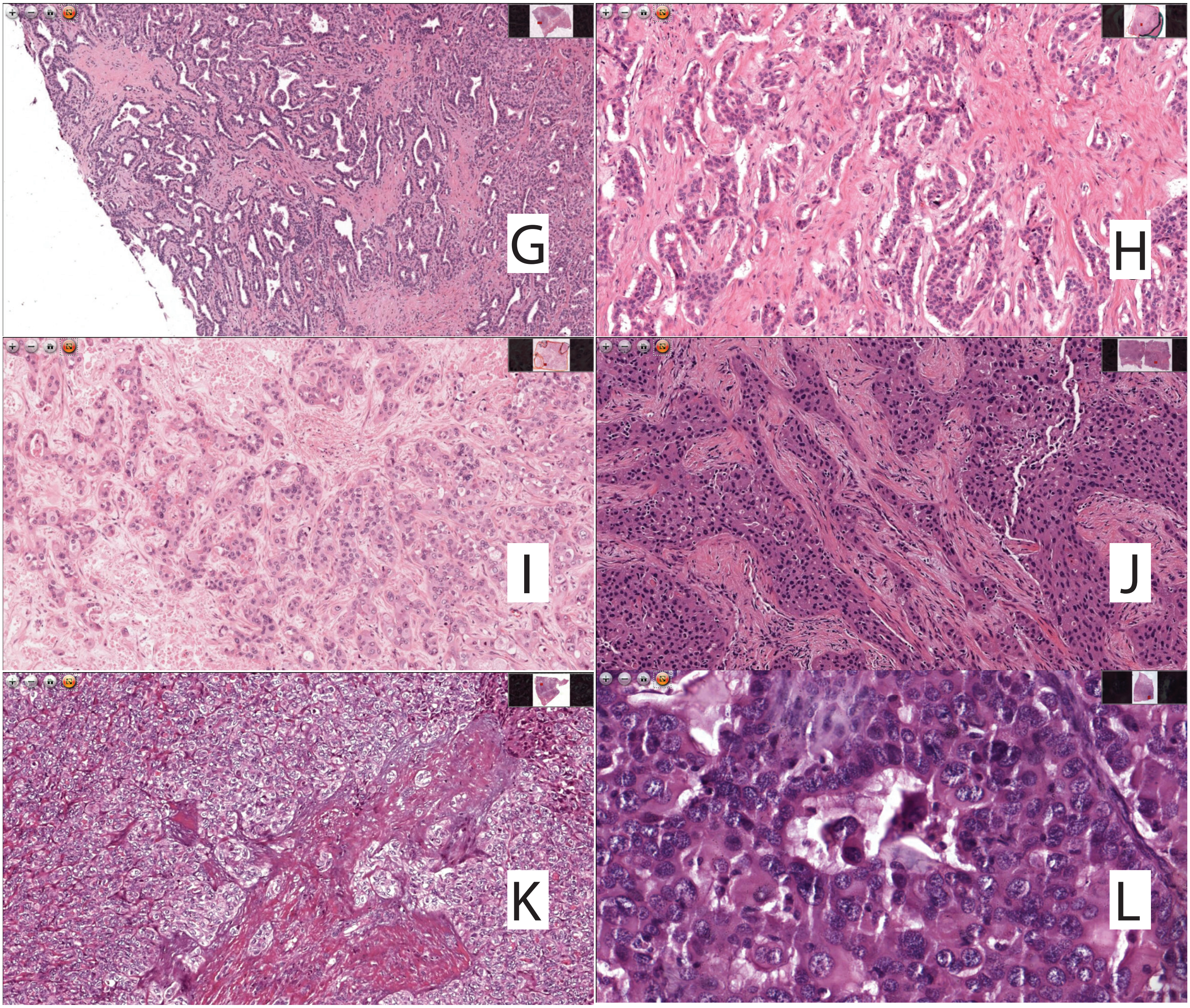
G
H
I
J
K
L

Supplemental Figure 6

M

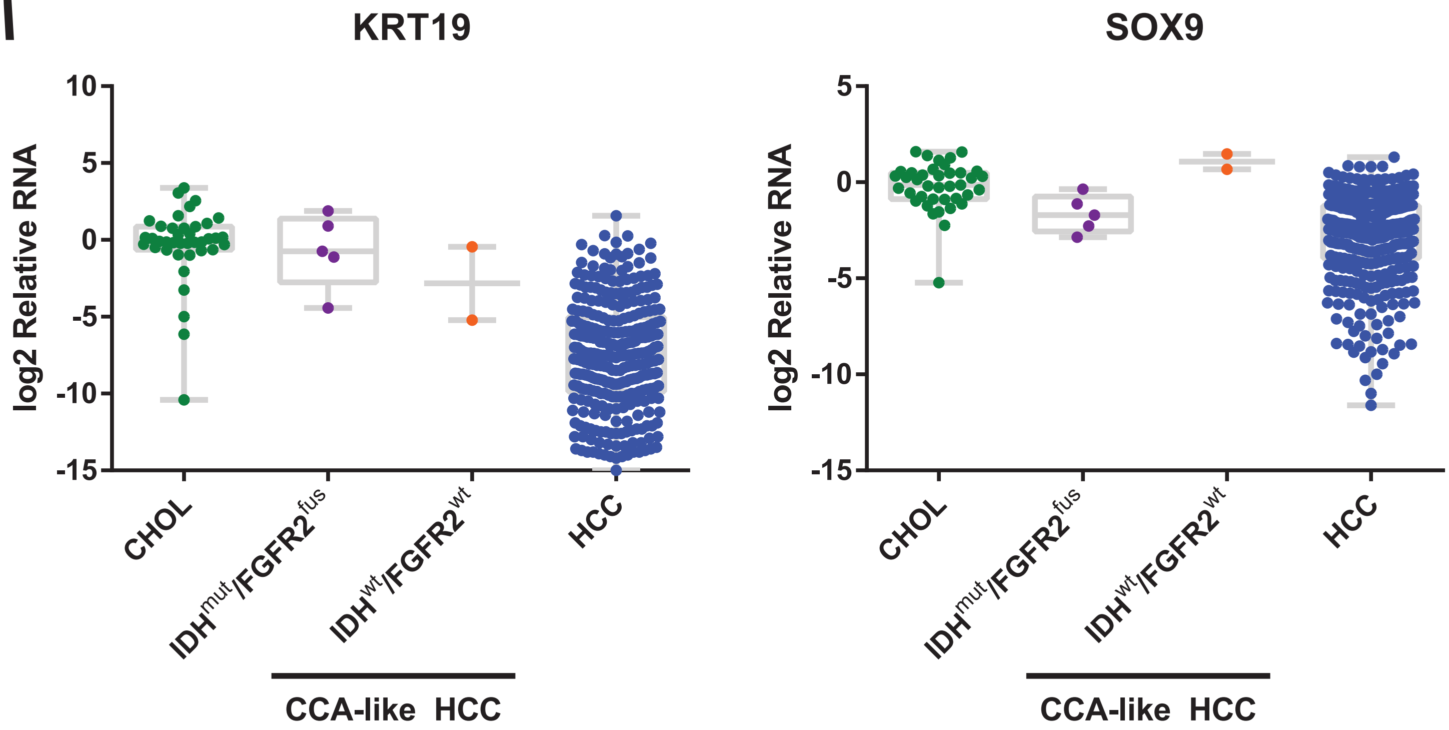

N

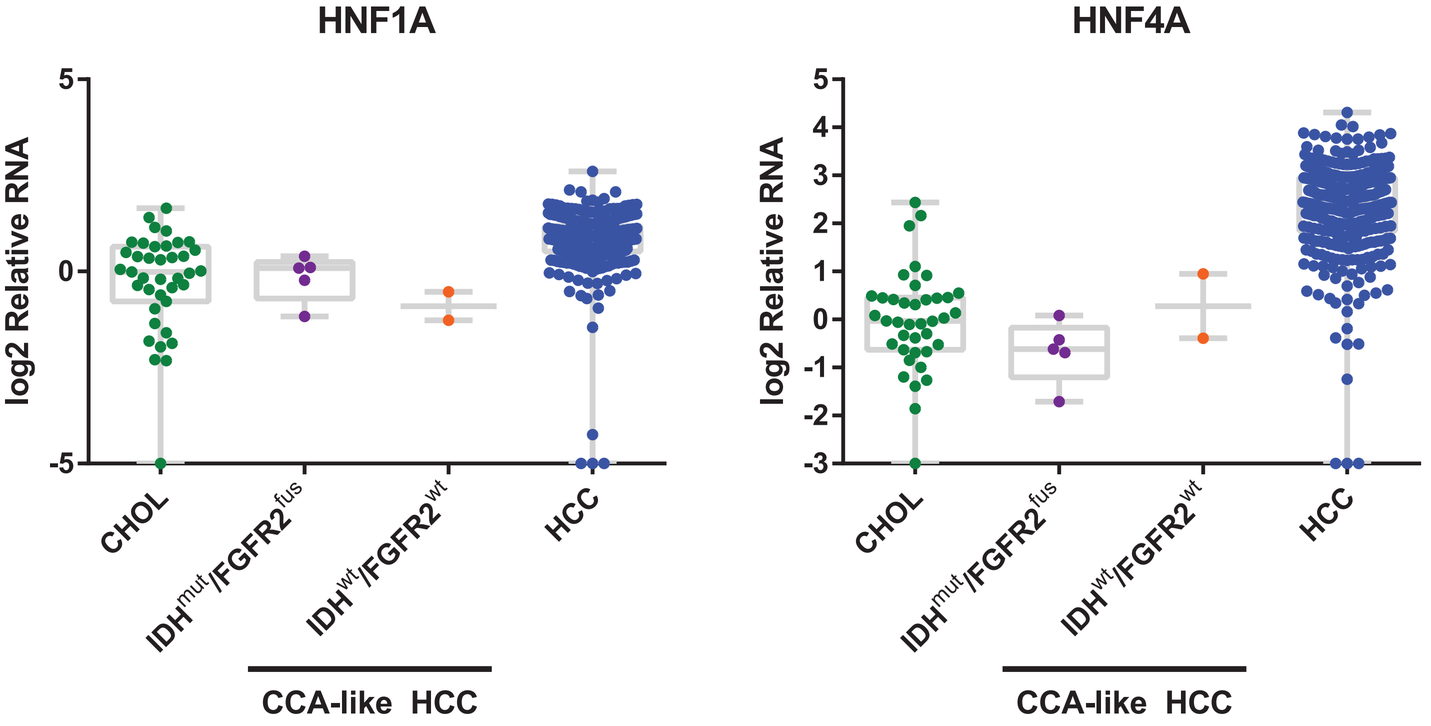

Supplemental Figure 6. Three-way cancer comparison analyses among HCC, PDAC, and CCA, related to Figure 6. A-F) Hierarchical clustering of 292 combined CCA, HCC, and PDAC samples from TCGA. A) DNA methylation. B) MicroRNA mature strands. C) Somatic copy number. D) Reverse-phase protein array. E) TumorMap analysis of mRNA. F) Hierarchical clustering of mRNA based on the TumorMap output. G-L) The most unusual histological characteristics from the group of 7 cholangiocarcinoma-like hepatocellular carcinomas. The tumor cells show varying amount of abundant eosinophilic cytoplasm. G) Extensive gland formation with a prominent anastomosing growth pattern (TCGA-G3-A25T). H) Focal pseudogland formation and an anastomosing pattern of growth as highlighted by the interconnecting character of the pseudoglands (TCGA-ED-A82E). I and J) Subtle evidence of an anastomosing pattern in I) TCGA-CD-A7PX and J) TCGA-DD-A4NA. K) Tumor with a nested pattern of growth (TCGA-CC-A3MA). L) Focal pseudoglandular differentiation (TCGA-CC-5260). M and N) Genes that are markers of cholangiocarcinoma and hepatocellular carcinoma are enriched in the genes that identify CCA-like HCCs. M) CCA marker genes *KRT19* and *SOX9*. N) HCC marker genes *HNF1A* and *HNF4A*.
